# Differential Modulation of Photosynthesis, Signaling, and Transcriptional Regulation between Tolerant and Sensitive Tomato Genotypes under Cold Stress

**DOI:** 10.1371/journal.pone.0050785

**Published:** 2012-11-30

**Authors:** Hui Liu, Bo Ouyang, Junhong Zhang, Taotao Wang, Hanxia Li, Yuyang Zhang, Chuying Yu, Zhibiao Ye

**Affiliations:** Key Laboratory of Horticultural Plant Biology, Ministry of Education, Huazhong Agricultural University, Wuhan, China; Kansas State University, United States of America

## Abstract

The wild species *Solanum habrochaites* is more cold tolerant than the cultivated tomato (*S. lycopersicum*). To explore the mechanisms underlying cold tolerance of *S. habrochaites*, seedlings of *S. habrochaites* LA1777 introgression lines (ILs), as well as the two parents, were evaluated under low temperature (4°C). The IL LA3969 and its donor parent LA1777 were found to be more cold tolerant than the recurrent parent *S. lycopersicum* LA4024. The differences in physiology and global gene expression between cold-tolerant (LA1777 and LA3969) and -sensitive (LA4024) genotypes under cold stress were further investigated. Comparative transcriptome analysis identified 1613, 1456, and 1523 cold-responsive genes in LA1777, LA3969, and LA4024, respectively. Gene ontology (GO) term enrichment analysis revealed that more GO biological process terms were significantly enriched among the up-regulated genes in the two tolerant genotypes, whereas more biological processes were significantly repressed by cold stress in the sensitive one. A total of 92 genes with significant differential expression between tolerant and sensitive genotypes under cold stress were identified. Among these, many stress-related GO terms were significantly enriched, such as ‘response to stimulus’ and ‘response to stress’. Moreover, GO terms ‘response to hormone stimulus’, ‘response to reactive oxygen species (ROS)’, and ‘calcium-mediated signaling’ were also overrepresented. Several transcripts involved in hormone or ROS homeostasis were also differentially expressed. ROS, hormones, and calcium as signaling molecules may play important roles in regulating gene expression in response to cold stress. Moreover, the expression of various transcription factors, post-translational proteins, metabolic enzymes, and photosynthesis-related genes was also specifically modulated. These specific modifications may play pivotal roles in conferring cold tolerance in tomato. These results not only provide new insights into the molecular mechanisms of cold tolerance in tomato, but also provide potential candidate genes for genetic improvement.

## Introduction

Low temperature is one of the key limiting factors that affect plant distribution, growth, development, production, and survival. Plants from temperate regions can increase freezing tolerance by cold acclimation [1], [2]. By contrast, plants from tropical regions, such as rice, maize, and tomato, are unable to cold acclimate and are sensitive to chilling temperatures (0 to 12°C) [3]. The cultivated tomato (*S. lycopersicum*) suffers from chilling injury at all stages of plant growth and development, including seed germination, growth, and fruit set. By contrast, the wild species *S. habrochaites* grows well at chilling temperatures and has been proposed as a potential germplasm resource of cold tolerance in tomato breeding [4]–[6]. In the past decade, many physiological responses to cold stress were comparatively analyzed between *S. habrochaites* and *S. lycopersicum*, for review see [5]. Although several hypotheses have been proposed to explain tolerance or sensitivity to chilling in plants, the physiological mechanisms responsible for cold tolerance remain unclear [7].

The C-repeat binding factor (CBF) cold response pathway is currently the best documented genetic system that plays a pivotal role in gene regulation during cold acclimation [2], [8]. Exposing *Arabidopsis* plants to low temperatures results in the rapid induction of *CBF* genes [9]. CBFs can bind to the CRT/DRE regulatory element in the promoters of many cold-inducible genes and activate their expression [2], [9], [10]. Global transcriptome analysis revealed that approximately 12% of cold-regulated genes are controlled by the CBFs in *Arabidopsis* [11]. Tomato also has a complete CBF cold response pathway, but its CBF regulon differs from that of *Arabidopsis* and appears to be considerably smaller and less diverse in function [3]. The CBF cold response pathway in tomato is not as important as in cold-acclimated plants.

Several studies have explored the genetic basis of cold tolerance in *S. habrochaites*. Vallejos and Tanksley identified three quantitative trait loci (QTLs) for the plastochron index at low temperatures using a BC1 population derived from cold-sensitive *S. lycopersicum* cv. T3 and a cold-tolerant *S. habrochaites* [12]. In another study, several QTLs associated with shoot wilting and root ammonium uptake under low temperatures were identified in a *S. lycopersicum × S. habrochaites* BC1 population [13]. A major-effect QTL for shoot turgor maintenance under root chilling stress was then fine mapped to a 2.7 cM region between markers T1670 and T1673 on chromosome 9 [7]. Meanwhile, an IL population of *S. habrochaites* LA1777 was developed by Monforte and Tanksley [14]. This IL population represents more than 85% of the genome of *S. habrochaites* LA1777 in the genetic background of *S. lycopersicum* LA4024. This IL population provides a valuable resource for exploring QTLs/genes involved in cold tolerance in tomato.

Transcriptome analysis using microarray has been widely used to investigate global gene expression in response to abiotic stress. Transcriptional profiling under cold stress has been carried out in different plant species, such as *Arabidopsis* [11], [15], rice [16], [17], and barley [18], [19]. In tomato, transcriptome analysis has been used to compare patterns of gene expression under salt or drought stress [20]–[22]. However, to the best of our knowledge, comparative transcriptome analysis of cold-tolerant and -sensitive tomato under cold stress is yet to be reported.

In this study, the IL LA3969 and its donor parent LA1777 were found to be more tolerant to cold stress than the recurrent parent *S. lycopersicum* LA4024 under cold stress. To explore the differences in gene expression between cold-tolerant and -sensitive tomato genotypes under cold stress, the gene expression profiles of the two tolerant genotypes (LA1777 and LA3969) and the cold-sensitive recurrent parent (LA4024) were comparatively analyzed. The results presented here provide new insights into the molecular mechanisms underlying the cold tolerance of the wild tomato *S. habrochaites*.

## Materials and Methods

### Plant Materials and Cold Stress Treatment

Seeds of 93 ILs as well as their two parents, *S. lycopersicum* LA4024 and *S. habrochaites* LA1777, were kindly supplied by Tomato Genetics Research Center (University of California, Davis, USA). Most of the lines contain a single defined introgression from LA1777 in the genetic background of LA4024 [14]. All the seeds were surface-sterilized and then sown individually in 10 cm diameter plastic pots containing peat, vermiculite and soil (v/v/v = 1∶1:1). The seedlings were grown in temperature regimes of 24–28°C day/20–25°C night and relative humidity of 70–80% under natural *light* in a greenhouse. Six-week-old seedlings were used for cold stress treatments.

To screen cold-tolerant ILs, uniform-sized plants were selected and transferred into a cold chamber with a temperature of 4°C, a 16 h photoperiod (irradiation intensity 120 µmol m^–2^s^–1^), and 70% to 80% relative humidity. Three replicates were used for each IL, with nine plants per replicate. In the first round of screening, 22 ILs that exhibited less severe wilting than the recurrent parent LA4024 after 3 d of chilling stress were identified as putative cold-tolerant lines. These selected ILs and the two parents were further analyzed. After two weeks of chilling stress and then one week of recovery, the survival rates of these ILs were recorded.

For physiological and microarray analysis, uniform seedlings of the selected line LA3969 and the two parents were used. The cold stress treatment was conducted as described above. Seedlings of the control group were grown at 25°C. The second and third leaves were sampled after 0, 1, 3, 5, and 7 d of treatment. The leaf samples were immediately frozen in liquid nitrogen and stored at −80°C until use. Three independent biological samples for each treatment were harvested, and each replicate contained 15 plants.

### Measurements of Electrolyte Leakage, Lipid Peroxidation, Proline and Total Soluble Sugars

Relative electrolyte leakage and lipid peroxidation were used to evaluate the cell membrane damage. Lipid peroxidation was estimated by determining malondialdehyde (MDA) content. Relative electrolyte leakage of leaf discs and MDA content were determined as described by Campos et al. [23]. Levels of free proline were measured according to the method described by Zhang et al. [24]. Total soluble sugar content was analyzed using the anthrone method, with glucose as the standard [25].

### Measurement of Chlorophyll Fluorescence Parameters

Chlorophyll fluorescence parameters of the third leaves were measured at different time points after the treatment using a pulse-modulated fluorometer (FMS-2, Hansatech, UK) according to the method described by Wingler et al. [26]. The maximum quantum efficiency of photosystem II (PSII) photochemistry was calculated as *F*v/*F*m = (*F*m-*F*_0_)/*F*m. The quantum yield of PSII electron transport, ΦPSII was calculated as ΦPSII = (*F*’m-*F*s)/*F*’m.

### Histochemical Detection of ROS

Histochemical staining of superoxide radical (O_2_^-^) and hydrogen peroxide (H_2_O_2_) was performed as previously described by Xia et al. [27] with minor modifications. The terminal leaflets of the first fully expanded leaf from six-week-old seedlings treated at 4°C for 0, 1, and 3 d were used for staining. To detect the presence of O_2_^-^, the leaflets were vacuum infiltrated in 50 mM potassium phosphate buffer (pH 7.8) containing 0.1 mg mL^−1^ nitroblue tetrazolium (NBT) and incubated at 25°C in the dark for 2 h. To detect the presence of H_2_O_2_, the leaflets were vacuum infiltrated in 1 mg mL^−1^ diaminobenzidine (DAB) in 50 mM Tris-acetate (pH 3.8) and incubated at 25°C in the dark for 8 h. To remove chlorophylls, the stained samples were transferred to 80% ethanol and incubated at 70°C for 10 min. Images were taken with a digital camera.

### Enzyme Extraction and Assay

Tomato leaves (approximately 200 mg) were homogenized in 2 mL of ice-cold 0.1 M phosphate buffer (pH 7.0) containing 0.1% polyvinylpyrrolidone. The homogenates were centrifuged at 4°C for 15 min at 12,000 rpm. The supernatant was used for the determination of enzyme activities. Superoxide dismutase (SOD; EC 1.15.1.1), ascorbate peroxidase (APX; EC 1.11.1.11), and catalase (CAT; EC 1.11.1.6) activities were assayed as described by Mittova et al. [28]. *Peroxidase* (*POD*; EC 1.11.1.7) activity was assayed following the method described by Morohashi [29].

### Microarray Analysis

Total RNAs from the leaf samples of the control and cold stress treatment (3 d at 4°C) were used for microarray analysis. Microarrays were performed using the TOM2 Oligo microarray (http://ted.bti.cornell.edu/). Three independent biological replicates were applied for each pair of control and cold stress treatments. Dual channel microarray hybridization was carried out with a Cy3-labeled control sample and a Cy5-labeled cold stress-treated sample. RNA isolation, amplification, labeling, and array hybridizations were performed essentially as described by Gong et al. [21]. Arrays were scanned with a LuxScan 10KA confocal laser scanner (CapitalBio, China), and the raw data were extracted using LuxScan™ 3.0 software (CapitalBio, China). A Print-tip Lowess Normalization method was used to normalize the ratio values [30]. Normalized ratio data were then log_2_ transformed. To identify cold-responsive genes, data were analyzed with the SAM (Significance Analysis of Microarrays) package [31]. Genes with a q-value (false discovery rate) of less than 0.05 and a Log_2_ ratio (cold stress/control) more than 2 or less than −2 were considered as cold-responsive genes. To further identify cold-responsive genes with significant (p<0.05) differential expression between tolerant and sensitive genotypes, statistical comparisons between tolerant and sensitive genotypes were made by Student’s t test using SigmaPlot 12. The probe sequences of differentially expressed genes were retrieved from the Tomato Functional Genomics Database (http://ted.bti.cornell.edu). To determine the chromosomal location of these genes, the probe sequences were further used as query sequences for the BLASTN search against SGN tomato whole genome chromosome database (SL2.40; http://solgenomics.net/tools/blast/index.pl). Gene annotation and GO term enrichment analysis were performed using the Tomato Functional Genomics Database [32]. Identification of significantly altered biochemical pathways was performed using the Plant MetGenMAP system [33]. The MapMan software was employed to reveal the cold-responsive genes associated with photosynthesis, ROS, calcium regulation, transcription, and post-translational modifications [34]. Gene expression profiles were clustered using Genesis software [35]. The microarray data have been deposited in the Tomato Functional Genomics Database, accession number E060.

### Quantitative RT-PCR

Total RNA was isolated using the TRIzol reagent (Invitrogen, USA), and DNase I was used to clean out DNA. First-strand cDNA was synthesized from 3 µg of total RNA with oligo(dT) and MMLV reverse transcriptase (Toyobo, Osaka, Japan) according to the manufacturer’s instructions. Quantitative real-time PCR (qPCR) was carried out using the *LightCycler480* System (Roche) and SYBR® Premix Ex Taq™ (TaKaRa) according to the supplier’s manual. The PCR cycling conditions were as follows: 95°C for 1 min, followed by 40 cycles of 95°C for 5s and 60°C for 20 s. Melting curve was routinely performed after 40 cycles to verify primer specificity. Tomato elongation factor 1α (*EF1α*) was used as internal control for qPCR analysis [36]. The fold change in the expression of each gene was calculated using the ΔΔCT method [18]. The relative gene expression level was log_2_ transformed to make the data easily comparable with the array value. The primers used for this analysis were designed using the Primer3 software (http://frodo.wi.mit.edu/primer3). The primer sequences are listed in Table S1.

## Results and Discussion

### Identification of Cold-tolerant IL

**Figure 1:**
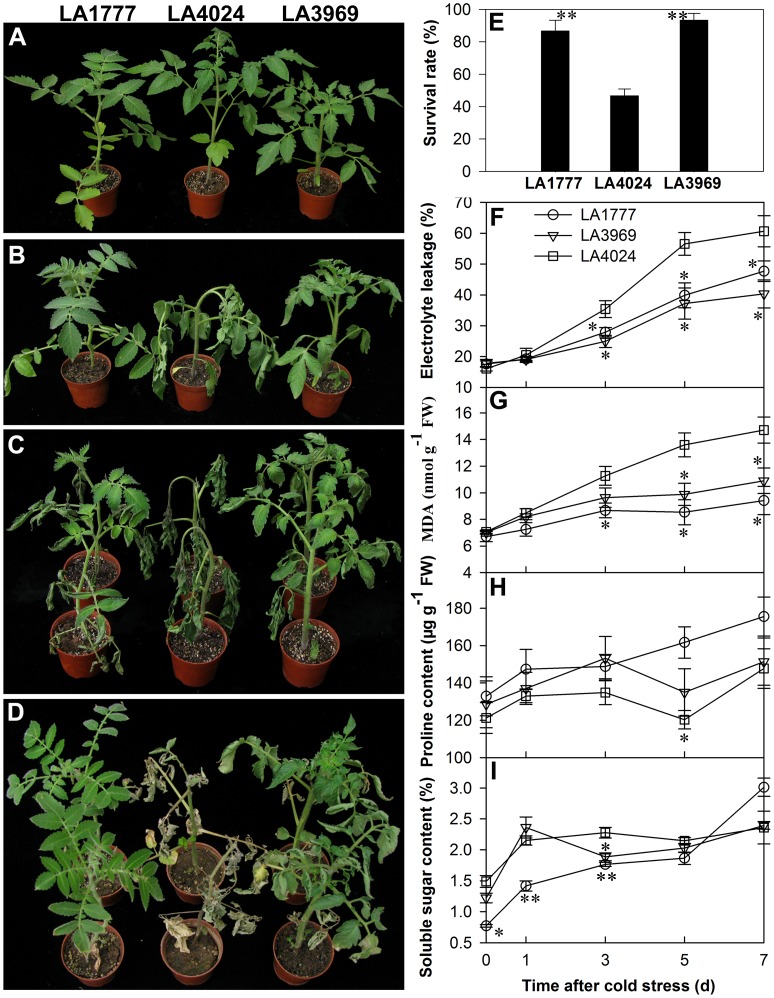
Phenotypic and physiological responses of the three tomato genotypes under cold stress. Seedlings of LA1777, LA4024, and LA3969 in control (A), treated at 4°C for 3 d (B), treated at 4°C for 10 d (C), and recovered for two weeks after10 d of cold treatment (D). Survival rates after two weeks of recovery (E). Changes in relative electrolyte leakage (F), MDA content (G), proline content (H), and soluble sugar content (I) in leaves of the three genotypes treated with 4°C for 0, 1, 3, 5, and 7 d. Three independent biological replicates were used in each treatment, with 15 plants (six-week-old) per replicate. Data are presented as mean ± SE of three independent biological replicates. Asterisks indicate statistically significant differences between tolerant and sensitive genotypes. *, p<0.05; **, p<0.01, Student’s t test.

To identify the major chromosomal regions of *S. habrochaites* genome conferring cold tolerance, seedlings of 93 *S. habrochaites* ILs and the two parents, *S. lycopersicum* LA4024 and *S. habrochaites* LA1777, were evaluated by cold stress treatment. Twenty-two ILs with *S. habrochaites* introgressions on chromosomes 1, 2, 3, 4, 5, 6, 7, 9, 11, and 12, exhibited less severe wilting than the recurrent parent LA4024 after 3 d of treatment at 4°C. After further treatment for two weeks, only one IL (LA3969) survived and exhibited strong tolerance to cold stress like the donor parent LA1777. To evaluate accurately the tolerance of LA3969, we compared the performance of this IL to its parents during cold stress. The phenotypic performance of LA3969 was quite close to that of LA1777 during cold stress and recovery, and showed stronger cold tolerance than LA4024 (Figures 1A to 1D). After 10 d of cold stress and recovery for two weeks, the survival rates of LA3969 and LA1777 were significantly higher than that of LA4024 (p<0.01; Figure 1E). Although some plants of LA4024 still survived, nearly all leaves were dead, except the newly grown ones (Figure 1D).

LA3969 contains a large introgressed segment from *S. habrochaites* on chromosome 12. Vallejos and Tanksley identified three QTLs from *S. habrochaites* responsible for growth at low temperatures, and the locus *Pgi-1* on chromosome 12 elicits a significant and positive effect only at low temperatures [12]. Truco et al. also detected a QTL from *S. habrochaites*, on chromosome 12, responsible for recovery from chill-induced wilting [13]. Therefore, at least one major QTL/gene responsible for cold tolerance is located on *S. habrochaites* chromosome 12.

### Less Severe Membrane Damage in the Cold-tolerant Genotypes

Plants subjected to low temperatures frequently suffer membrane damage, which can be evaluated by the relative electrolyte leakage and MDA production [23]. During cold stress, all tomato genotypes presented a tendency to increase the relative electrolyte leakage and MDA content, but the levels of the two tolerant genotypes were significantly lower than that of the sensitive one after 3 d of cold stress (p<0.05; Figures 1F and 1G). The results indicated that the cold-tolerant genotypes suffer less severe membrane damage than the sensitive genotype under cold stress.

Compatible solutes, such as proline and carbohydrates, play important roles in cell osmotic adjustment and maintaining membrane integrity [37]. Therefore, we analyzed the differences in accumulation of proline and soluble sugars between cold-tolerant and -sensitive genotypes under cold stress. During cold stress, the proline content increased continuously in LA1777. After 5 d of cold treatment, LA1777 had significantly (p<0.05) higher level of proline than LA4024. But no significant difference in proline content was found between LA3969 and LA4024 during cold stress (Figure 1H). The total soluble sugar content exhibited an upward trend in all three genotypes during cold stress. Only at 3 d of cold treatment, both tolerant genotypes showed significantly (p<0.05) lower levels of total soluble sugar than the sensitive one (Figure 1I). Therefore, a positive correlation between proline and soluble sugars accumulation and cold tolerance in tomato could not be found.

### Differences in Gene Expression between Tolerant and Sensitive Genotypes under Cold Stress

**Figure 2:**
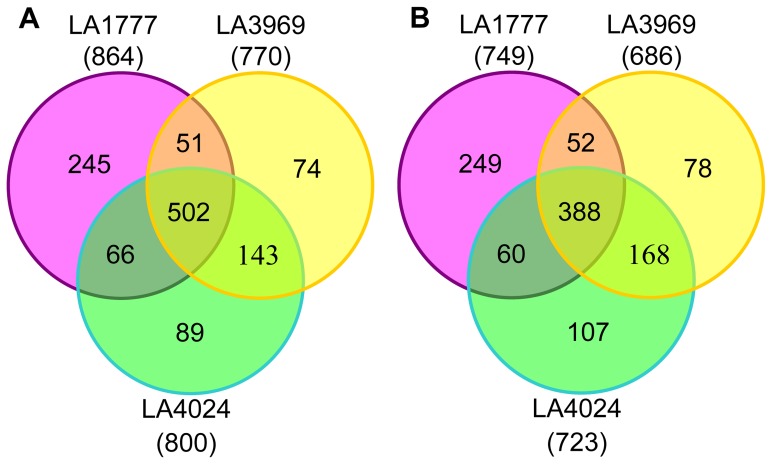
Venn diagrams showing number and overlap of differentially expressed genes under cold stress in LA1777, LA3969, and LA4024. (A) Number of up-regulated genes (log_2_ ratio stress/control≥2 and q-value<0.05). (B) Number of down-regulated genes (log_2_ ratio stress/control≤−2 and q-value<0.05). The number in parentheses indicates the total number of genes up- or down-regulated by cold stress in each genotype.

To investigate the differences in gene expression between cold-tolerant (LA1777 and LA3969) and -sensitive (LA4024) tomato genotypes in response to cold stress, we performed comparative transcriptome analysis using TOM2 microarray. After 3 d of cold stress, a total of 1613 (864 up- and 749 down-regulated), 1456 (770 up- and 686 down-regulated), and 1523 (800 up- and 723 down-regulated) cold-responsive genes (q-value<0.5, log_2_ ratio (cold stress/control) above 2 and below -2) were identified in LA1777, LA3969, and LA4024, respectively (Figures 2A and 2B). Among them, 103 cold-responsive genes (51 up- and 52 down-regulated) were exclusively identified in both LA1777 and LA3969, whereas 196 cold-responsive genes (89 up- and 107 down-regulated) were uniquely observed in LA4024. A total of 890 genes (502 up- and 388 down-regulated) were commonly regulated by cold stress in all three tomato genotypes. The large number of cold-responsive genes identified in all three genotypes suggests a common response mechanism to cold stress between cold-tolerant and -sensitive genotypes. *S. habrochaites* LA1777 showed a higher number of specific cold-responsive genes compared to LA3969 and LA4024 (Figures 2A and 2B). These genes may reveal the difference in response to cold stress between *S. habrochaites* and *S. lycopersicum*.

**Figure 3:**
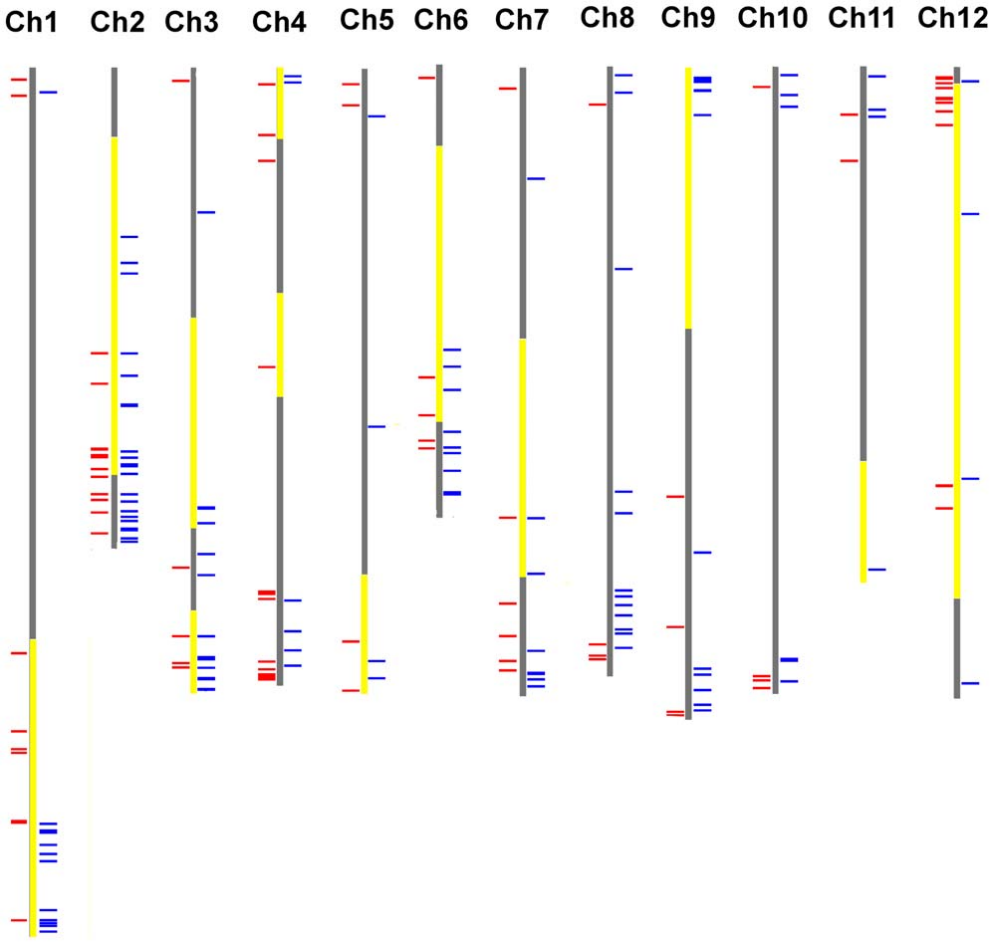
Chromosomal distribution of genes differentially expressed between the tolerant and sensitive genotypes under cold stress. Each horizontal line represents one gene. The red lines represent the 92 genes (as shown in Figures S1) with significant difference in expression between the two tolerant and sensitive genotypes under cold stress. The blue lines represent the 126 genes (as shown in Table S2) whose expression level in LA1777 is significantly different from that in LA3969 and LA4024. The yellow regions on chromosomes indicated the introgressed chromosomal regions of the 22 selected cold-tolerant ILs and/or cold tolerance QTLs identified previously in *S. habrochaites* [7], [12], [13]. Chromosome numbers are indicated at the top of each bar. Question mark indicates probe sequence dose not match on chromosome.

To distinguish the cold-responsive genes which are differentially expressed between tolerant and sensitive genotypes under cold stress, the individual gene expression levels of tolerant and sensitive genotypes were compared further by using Student’s t test. A total of 92 cold-responsive genes with statistically significant differences (p<0.05) in expression between tolerant and sensitive genotypes were identified (Figure S1). To identify genes that localize to genomic regions contributing to cold tolerance, these 92 genes were mapped to the tomato chromosomes (SL2.40). Thirty-two genes were found to be located on the introgressed chromosomal segments of the 22 selected cold-tolerant ILs and/or cold tolerance QTLs identified previously in *S. habrochaites* (Figures 3 and S1). Of these, 11 genes were localized to the introgressed segment of LA3969. According to expression profiles and gene annotation, five genes may play critical roles in conferring the difference in cold tolerance between LA3969 and LA4024. Among them, four (SGN-U212650, SGN-U219719, SGN-U212639, and SGN-U216055) were more strongly induced by cold stress in the tolerant genotypes than in the sensitive one (Table 1). SGN-U212650 encoding leucine aminopeptidase A1 (LAP-A1) was up-regulated by 4.04-, 3.96-, and 1.70-fold in LA1777, LA3969, and LA4024, respectively. *LAP-A1* transcript is induced by various stimuli, and it is essential for regulating defense and wound signaling in tomato [38], [39]. SGN-U216055 encodes a NAC transcription factor similar to *Arabidopsis* RD26. The expression of *RD26* is induced by cold in *Arabidopsis*, and its transcript level in response to low temperature is significantly reduced in the *pi4kIIIβ1β2* double mutant whose germination is hypersensitive to chilling [40]. SGN-U212639 encodes a protein similar to molecular chaperone Hsp90-1. HSP90 is recruited for stomatal closure and serves essential functions in plants to integrate signals from their biotic and abiotic environments [41]. Glutathione plays a critical role in maintaining cellular homeostasis and is essential for the regulation of oxidant stress. The key enzyme involved in glutathione homeostasis is gamma-glutamyltransferase. SGN-U218110 encoding a gamma-glutamyltransferase was more severely repressed in LA4014 than in LA1777 and LA3969 under cold stress (Table 1).

**Table 1:** Top 30 differentially expressed genes most likely to confer cold tolerance in *S. habrochaites* LA1777 and their corresponding gene expression among the three genotypes under cold stress.

No.	Unigene ID	Relative expression level^A^			Chr^B^	Annotation
		LA1777	LA3969	LA4024		
1	SGN-U212650	4.04	3.96	1.70	12	Leucine aminopeptidase 1
2	SGN-U219719	4.26	3.05	1.86	12	Hypothetical protein
3	SGN-U212639	2.64	2.16	1.38	12	Molecular chaperone Hsp90-1
4	SGN-U218110	−1.72	−1.74	−2.86	12	Gamma-glutamyltransferase
5	SGN-U216055	3.39	3.70	2.55	12	Jasmonic acid 2 (RD26)
6	SGN-U215018	−0.39	−0.69	−3.20	6	Hypothetical protein
7	SGN-U226166	2.26	2.18	−0.43	10	Glutathione S-transferase
8	SGN-U221957	0.22	−0.72	−2.58	7	Heat stress transcription factor
9	SGN-U213712	−0.59	−1.71	−2.24	1	Beta-amylase
10	SGN-U214930	3.11	2.77	0.96	2	Hypothetical protein
11	SGN-U215106	2.25	2.78	0.95	4	SKP2A
12	SGN-U234004	2.41	2.86	4.34	7	MYB15
13	SGN-U239712	3.04	2.66	1.88	5	Phenylalanine ammonialyase 1
14	SGN-U220721	5.18	3.60	2.52	1	Ca2+-binding protein
15	SGN-U215123	4.07	4.74	5.62	2	WRKY protein
16	SGN-U212850	−1.02	6.03	5.58	9	Peroxidase
17	SGN-U227893	0.55	−3.35	−3.26	9	Amine oxidase
18	SGN-U212825	3.37	0.68	0.88	9	LOS2
19	SGN-U213115	2.83	−1.89	−1.97	1	Hypothetical protein
20	SGN-U242106	−4.43	0.64	−0.04	2	UDP-glycosyltransferase
21	SGN-U213791	−0.24	3.21	2.58	2	Acidic 27 kDa endochitinase
22	SGN-U214065	−0.09	2.97	2.47	1	UDP-glucosyltransferase
23	SGN-U216044	0.08	−2.51	−3.05	1	Hypothetical protein
24	SGN-U229565	−2.65	0.18	0.23	2	Zinc finger family protein
25	SGN-U214425	−2.84	−0.39	−0.15	5	Ripening regulated protein
26	SGN-U223737	2.74	0.05	0.08	11	Aldehyde oxidase 1 homolog
27	SGN-U216449	2.27	0.61	0.78	3	PENETRATION 3
28	SGN-U218236	2.93	1.44	0.89	6	Hypothetical protein
29	SGN-U216729	−3.07	−1.62	−1.97	4	MAP KINASE
30	SGN-U223072	3.71	1.46	2.00	7	Calmodulin-binding protein

The numbers from 1 to 15 are the top 15 genes with significant difference in expression between the two tolerant and sensitive genotypes at 3 d of cold treatment (4°C). The numbers from 16 to 30 are the top 15 genes whose expression levels in LA1777 are significantly different from that in LA3969 and LA4024 under cold stress. (A) Positive (up-regulated) and negative (down-regulated) expression values (log_2_ ratio cold stress/control) are means of three independent biological replicates. (B) Chr, chromosomal localization.

According to chromosomal location, expression profiles, and gene annotation, ten genes located on other chromosomes may also confer cold tolerance in LA3969 (Table 1). Among these, SGN-U213712, SGN-U215018, and SGN-U221957 coding for a beta-amylase, a hypothetical protein, and a heat stress transcription factor, respectively, were markedly suppressed in LA4024. SGN-U215123 and SGN-U234004 encoding homologues of *Arabidopsis* WRKY31 and MYB15 were more strongly induced by cold stress in LA4024 than in LA1777 and LA3969. *AtMYB15* has been found to negatively regulate the expression of cold-responsive genes in *Arabidopsis* [42]. The other five genes were more strongly induced in the two tolerant genotypes than in the sensitive one. The precise roles of these differentially expressed genes remain to be elucidated via other experimental approaches, such as over-expression and/or RNAi strategies.

### Differences in Gene Expression between LA1777 and LA3969 under Cold Stress

LA3969 only contains a large introgressed segment from chromosome 12 of *S. habrochaites*, there are still additional QTLs for cold tolerance on other chromosomes of *S. habrochaites*. To further identify other genes which may confer cold tolerance in *S. habrochaites*, the expression levels of individual genes were compared further between LA1777 and LA3969 by using Student’s t test. A total of 295 cold-responsive genes with statistically significant differences (p<0.01) in expression between LA1777 and LA3969 were identified. Among these, 126 genes also showed statistically significant differences in expression between LA1777 and LA4024 (Table S2). Of these, 48 genes were mapped to the introgressed regions of the 22 selected cold-tolerant ILs and/or cold tolerance QTLs identified previously in *S. habrochaites* (Table S2). According to chromosomal location, expression profiles, and gene annotation, 15 genes that are most likely to confer cold tolerance were identified (Table 1). The expression of seven genes in LA3969 and LA4024 showed opposite expression patterns from LA1777 under cold stress. SGN-U214065, SGN-U229565, SGN-U213791, and SGN-U212850, coding for a UDP-glucosyltransferase, a zinc finger family protein, an acidic 27 kDa endochitinase, and a peroxidase, respectively, were down-regulated by cold stress in LA1777, whereas they were up-regulated in LA3969 and LA4024. For example, SGN-U212850 was down-regulated by 1.02-fold in LA1777, but it was induced by more than 5.50-fold in LA3969 and LA4024 under cold stress. On contrary, two hypothetical proteins (SGN-U213115 and SGN-U216044), and an amine oxidase (SGN-U227893) were up-regulated by cold stress in LA1777, while they were down-regulated in LA3969 and LA4024. Six genes (SGN-U215206, SGN-U216449, SGN-U218236, SGN-U223072, SGN-U212825, and SGN-U223737) showed significantly higher fold induction in LA1777 than in LA3969 and LA4024 under cold stress. It suggested these genes may play positive roles in response to cold stress in tomato. For instance, SGN-U212825 encoding a homologue of *Arabidopsis* LOS2 was up-regulated by 3.37-fold under cold stress in LA1777, whereas only by 0.67- and 0.88-fold in LA3969 and LA4024, respectively (Table 1). In *Arabidopsis*, *LOS2* encodes a bi-functional enolase and plays a positive role in the regulation of cold-responsive genes expression via transcriptional repression of *ZAT10*/*STZ* which is a negative regulator of CBF-target genes [43].

### Confirmation of Microarray Results

To confirm our microarray results, 42 genes with differential expression patterns were evaluated using qPCR. The microarray data showed a very good correlation with the qPCR results (*r* = 0.93; Figure S2). Most of these genes verified by qPCR showed the same expression patterns among the three tomato genotypes as in microarrays. Differences between the two methods were on the quantitative levels. The qPCR results showed a slightly higher fold induction or repression than the microarray analysis (Table S3). Similar phenomenon has been reported previously [44].

Previous studies have identified several genes that were regulated by cold stress in tomato. Among them, seven were identified as cold-responsive genes in microarray results. These include dehydrin [45], [46], *SAP8* and *SAP11* [47], alternative oxidase [48], *LeVDE* [49], *ERF2* [50], and *GME* [51]. The expression patterns of these genes were similar among the three genotypes or higher in the two tolerant genotypes (Table S4). For example, SGN-U213745 encoding a dehydrin protein was up-regulated significantly higher in the two tolerant genotypes than in the sensitive genotype when subjected to cold stress (4°C) for 3 days (p<0.05; Figure S1). This gene has been considered to be a marker gene for cold stress response in tomato [46]. These results demonstrate that our microarray results are reliable.

### GO Term Enrichment Analysis

**Figure 4:**
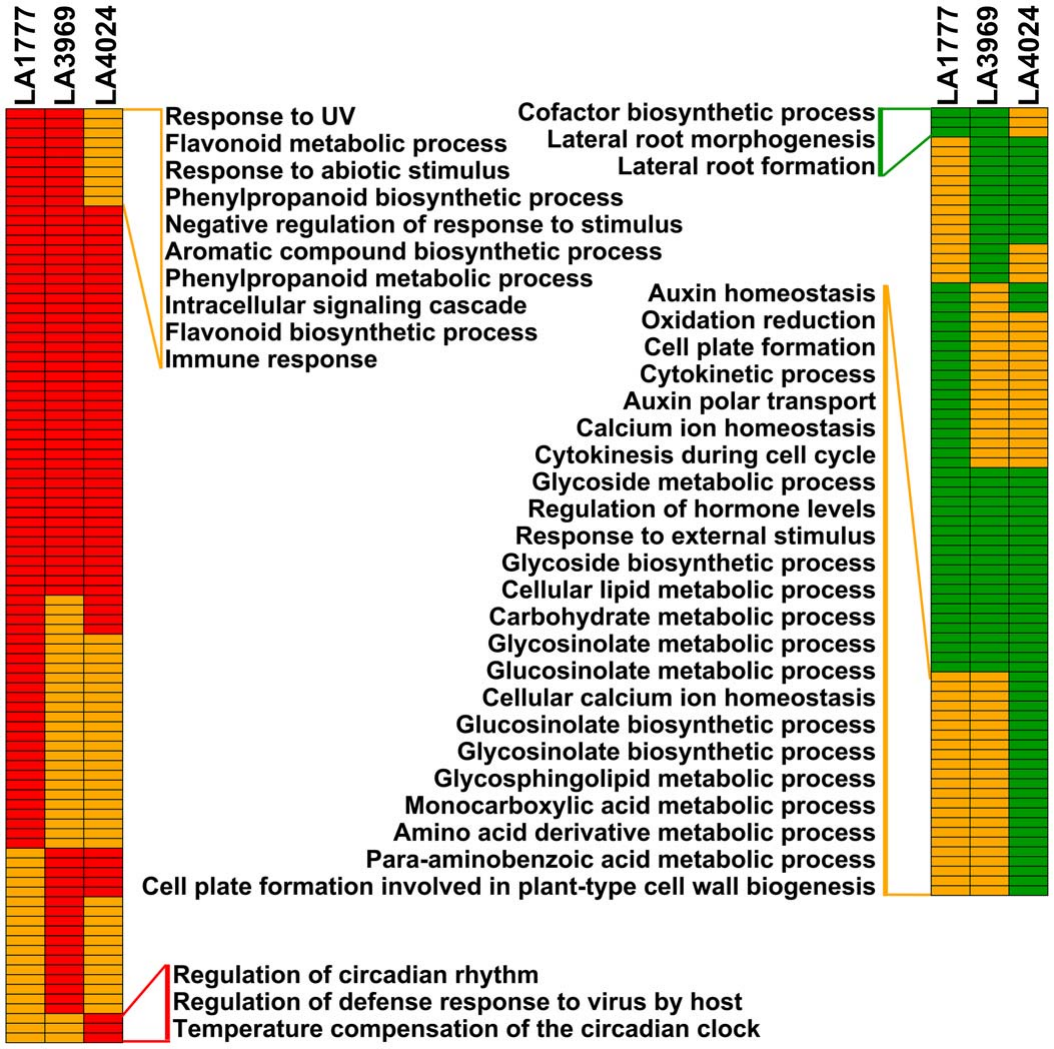
Comparison of GO biological process terms significantly enriched among the up- and down-regulated genes of LA1777, LA3969, and LA4024 under cold stress. The image was generated using Genesis software [35]. Each colour rectangle in the figure represents one GO term. Red indicates GO biological process terms that are significantly enriched (p<0.01, FDR as the cut off) among the up-regulated genes, green indicates GO biological process terms that are significantly enriched among the down-regulated genes, and yellow indicates no significant. Significantly enriched GO biological process terms identified in both tolerant genotypes or exclusively in the sensitive one are listed. For more details see Table S4.

To unravel the significantly altered biological processes upon cold stress, the up- and down-regulated genes of the three tomato genotypes were subjected to the GO term enrichment analysis [32]. As expected, some stress-related GO biological process terms, such as ‘response to stress’, ‘response to temperature stimulus’, ‘response to stimulus’, ‘response to abiotic or biotic stimulus’, and ‘defense response’, were significantly enriched (p<0.01, FDR as the cut-off) among the up- or down-regulated genes in at least one tomato genotype (Table S5). More GO terms were significantly enriched (p<0.01, FDR as the cut-off) among the up-regulated genes in the two tolerant genotypes (Figure 4; Table S5). Among these, ten GO terms were specially enriched in both tolerant genotypes. Of these, four were involved in stress responses, including ‘immune response’, ‘response to abiotic stimulus’, ‘negative regulation of response to stimulus response’, and ‘response to UV’. Only three GO terms were exclusively enriched among the up-regulated genes in LA4024. However, 23 biological processes were significantly suppressed only in LA4024 (Figure 4; Table S5). Interestingly, some of them are associated with stress responses, such as ‘response to external stimulus’, ‘regulation of hormone levels’, ‘calcium ion homeostasis’, and ‘oxidation reduction’ (Figure 4). More biological processes were significantly inhibited exclusively in LA4024 suggests the sensitive genotype is more severely influenced than the two tolerant genotypes under cold stress.

To explore the modulated biological processes between tolerant and sensitive genotypes under cold stress, the 92 differentially expressed genes were also analyzed using GO term enrichment analysis [32]. A total of 21 biological process GO terms varied significantly between tolerant and sensitive genotypes under cold stress (p<0.01, FDR as the cut-off; Table S6). Most of them were involved in stress responses, such as ‘response to stimulus’, ‘response to heat’, ‘response to abiotic stimulus’, ‘response to stress’, and ‘response to UV’. A large proportion (41.3%) of differentially expressed genes was classified into functional category ‘response to stimulus’. Moreover, GO terms ‘response to auxin stimulus’, ‘response to gibberellin stimulus’, ‘response to reactive oxygen species (ROS)’, ‘brassinosteroid metabolic process’, and ‘calcium-mediated signaling’ were also overrepresented. Several transcripts involved in hormone or ROS homeostasis were also found to be differentially expressed between tolerant and sensitive genotypes under cold stress. These results indicate ROS, hormones, and calcium as signaling molecules may play important roles in regulating gene expression in response to cold stress.

### Less Inhibition of Photosynthesis in the Cold-tolerant Genotypes

**Figure 5:**
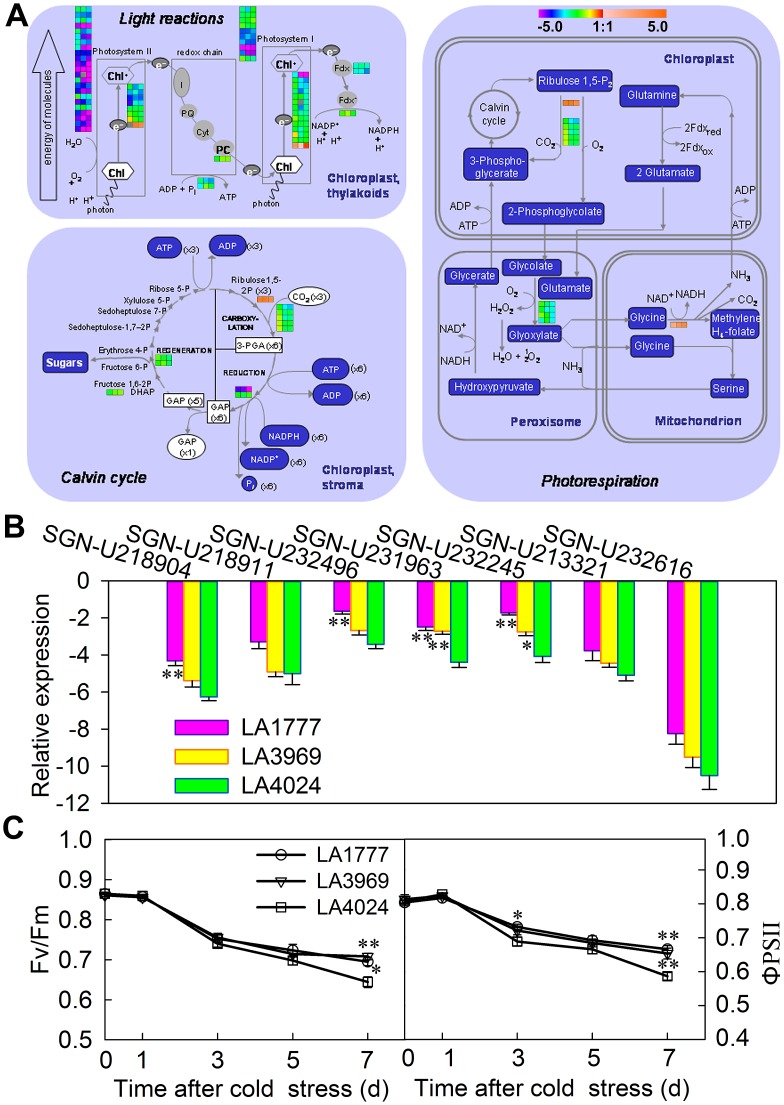
Comparison of the effects of cold stress on photosynthesis-related gene expression and chlorophyll fluorescence characteristics among the three tomato genotypes. (A) Differential expression of genes involved in photosynthesis among the three genotypes subjected to cold stress (4°C) for 3 d. This figure was modified from the photosynthesis pathway obtained using MapMan software [34]. The expression images were generated using Genesis software [35]. The three color squares from left to right indicate the expression levels of one related gene in LA1777, LA3969, and LA4024, respectively. The color intensity represents gene expression value (log_2_ ratio stress/control), as indicated by the color scale. Gray squares indicates the missing data. More details are given in Table S5. (B) qPCR analysis of selected photosynthesis-related gene expression under cold stress. Leaf samples from control and cold-treated (3 d at 4°C) plants were used for qPCR analysis. *EF1α* expression was used as internal control. The relative expression level is shown as log_2_ ratio (cold stress/control). Data are presented as mean ± SE of three independent biological replicates. Asterisks indicate a significant difference between tolerant and sensitive genotypes. *, p<0.05; **, p<0.01, Student’s t test. (C) Comparison of the maximum quantum efficiency of PSII photochemistry (*F*v/*F*m) and the quantum yield of PSII *electron* transport (ΦPSII) among the three genotypes during cold stress. Six-week-old seedlings were treated at 4°C for 0, 1, 3, 5, and 7 d. Measurements were performed on the third leaves of plants. Data are presented as mean ± SE of six replicates. Asterisks indicate a significant difference between tolerant and sensitive genotypes. *, p<0.05; **, p<0.01.

GO term enrichment analysis showed that photosynthesis was significantly inhibited by cold stress, and more related genes were down-regulated in LA4024 (Tables S5 and S7). The significant suppression of photosynthesis-related genes under cold stress has been observed in *Arabidopsis* and barley [15], [18]. The down-regulated genes covered all the aspects of photosynthesis, including the light reactions, Calvin cycle, and photorespiration (Figure 5A; Table S7). The suppression of the photosynthetic light reactions contained PSI, PSII, ATP synthase, and electron carriers. Many transcripts involved in PSII were strongly repressed in LA4024, and some were down-regulated by more than 5-fold, such as SGN-U218907, SGN-U218911, SGN-U234083, SGN-U212937, and SGN-U218904. Most of these genes encode chlorophyll a/b binding proteins. In addition, some genes involved in photorespiration and calvin cyle were also severely suppressed in LA4024, such as SGN-U232245, SGN-U212963, SGN-U215203, and SGN-U213321 (Table S7). A transcript, SGN-U225498, encoding RuBisCO small subunit 3B was more strongly induced in the two tolerant genotypes (Figure S1). The expression of seven genes involved in photosynthesis was validated by qPCR. As shown in Figure 5B, nearly all of them were strongly down-regulated in LA4024. Among these, SGN-U231963 and SGN-U232245 coding for a ferredoxin-NADP(+)-oxidoreductase and a glycolate oxidase, respectively, were less severely suppressed in LA1777 and LA3969 than in LA4024 (p<0.05). SGN-U218904 and SGN-U232496 coding for a chlorophyll a/b binding protein and a PSII reaction center W protein, respectively, were less severely suppressed in LA1777 than in LA4024 (p<0.01).

Transcriptome analysis suggested that the PSII was inhibited more severely in the sensitive genotype (Figure 5A; Table S7). To confirm this, the maximum quantum efficiency of PSII photochemistry (Fv/Fm) and the quantum yield of PSII electron transport (ΦPSII) were measured. As shown in Figure 5C, all tomato genotypes displayed a time-dependent decline in Fv/Fm and ΦPSII under cold stress, but the decrease was greater in the sensitive genotype. After 7 d of cold stress treatment, LA1777 and LA3969 showed significantly higher values of Fv/Fm and ΦPSII than LA4024. Previous study indicated that *S. habrochaites* showed less severe inhibition of photosynthesis than *S. lycopersicum* during chilling stress [4]. These results demonstrate that photosynthesis (especially PSII) is suppressed more severely in the cold-sensitive genotype than in the cold-tolerant genotype under cold stress.

### Differences in Gene Expression and Enzyme Activity Related to ROS between Tolerant and Sensitive Genotypes under Cold Stress

GO term enrichment analysis showed that the biological process ‘oxidation reduction’ was significantly suppressed in LA4024 (Figure 4), and the functional category ‘response to ROS’ was significantly enriched among the differentially expressed genes between tolerant and sensitive genotypes under cold stress (Table S6). To further characterize the differences in ROS-related gene expression between tolerant and sensitive genotypes, cold-responsive genes related to ROS were analyzed. A total of 74 ROS-related genes were regulated by cold stress, and eight of them were confirmed by qPCR (Table S8; Figure S3A). Among these, seven showed significant differences in gene expression between tolerant and sensitive genotypes under cold stress (Table S8). Both microarray and qPCR analysis showed that SGN-U215231 was more strongly induced by cold stress in LA1777 and LA3969 than in LA4024 (Figures S1 and Figure S3A). SGN-U215231 encodes a peroxidase similar to the pepper peroxidase CaPO2. qPCR analysis showed that SGN-U215628 encoding an amine oxidase was more severely repressed by cold stress in LA4024 than in LA3969 and LA1777 (Figure S3A). Both *CaPO2* and amine oxidase are involved in ROS generation [52], [53]. Three glutathione S-transferase genes (SGN-U213825, SGN-U214482, and SGN-U226166) were more strongly induced by cold stress in LA1777 and LA3969 than in LA4024 (Table S8). For instance, SGN-U226166 was up-regulated by 2.26- and 2.18-fold in LA1777 and LA3969, respectively, but it was down-regulated by 0.43-fold in LA4024 under cold stress. Previous studies demonstrated that overexpression of *GSTs* in tobacco improved cold tolerance [54], [55]. The significantly higher expression of *GSTs* in the two tolerant genotypes might have contributed to cold tolerance by reducing oxidative damage and regulating cellular redox homeostasis.

We further analyzed the activities of some antioxidant enzymes under cold stress, including APX, *POD*, CAT, and SOD. After 3 d of cold treatment, APX activity was significantly lower in the two tolerant genotypes than in the sensitive one (Figure S3B). By contrast, *POD* activity was significantly higher only at 3 d of cold treatment in the two tolerant genotypes than in the sensitive one (Figure S3C). This is consistent with the expression pattern of peroxidase gene (SGN-U215231) mentioned above. CAT activity was significantly decreased in LA1777 than in LA4024 after 5 d of cold treatment, but no significant difference was found between LA396 and LA4024 during cold stress (Figure S3D). No significant difference in SOD activity was found between tolerant and sensitive genotypes during cold stress (Figure S3E).

To check whether the changes in gene expression and enzyme activities would cause differences in ROS accumulation between tolerant and sensitive genotypes under cold stress, the presence of H_2_O_2_ and O_2_^-^ in the leaves of the three genotypes were detected with DAB and NBT staining. All three tomato genotypes exhibited a tendency of increased staining, indicating the accumulation of ROS during cold stress. But no remarkable difference between tolerant and sensitive genotypes was observed (Figures S3F and S3G). ROS have traditionally been regarded as toxic molecules. However, recent studies indicated that ROS play a key role in the complex signaling networks in plant [56]–[58]. Transcriptome analysis revealed that oxidative-mediated transcriptional regulatory network configures the early response mechanisms to chilling stress in japonica rice [17]. Thus, the accumulation of ROS may not be the main reason that causes cellular damage under cold stress in tomato. ROS as signaling molecules may play a critical role in regulating gene expression in response to cold stress.

### Differentially Expressed Genes Involved in Hormone Metabolism and Signaling

Comparative transcriptome analysis revealed that a large number of genes related to abscisic acid (ABA), jasmonic acid (JA), auxin, cytokinins (CKs), ethylene, gibberellin, and brassinolides were regulated by cold stress in tomato (Table S9). Three hormone-related GO biological processes were significantly enriched among the differentially expressed genes between tolerant and sensitive genotypes under cold stress, which are ‘response to auxin stimulus’, ‘response to gibberellin stimulus’, and ‘brassinosteroid metabolic process’ (Table S6). Hormones as signaling molecules are supposed to play important roles in regulating gene expression in response to cold stress in tomato.

A total of 14 hormone-related genes were differentially expressed between tolerant and sensitive genotypes under cold stress (Table S9). Among these, SGN-U214274 encoding a homologue of *Arabidopsis* ABA3 was more severely suppressed in the sensitive genotype, which was validated by qPCR (Figure S1; Table S3). In *Arabidopsis*, the ABA-deficient mutant aba3/los5 shows a significant reduction in the expression of cold stress-responsive genes and exhibits higher sensitivity to freezing stress [59]. The significant suppression of this gene may influence the expression of many downstream cold-responsive genes in the sensitive genotype. Two auxin-related genes, SGN-U215655 and SGN-U215106, were differentially expressed between tolerant and sensitive genotypes under cold stress. SGN-U215655 encoding an auxin-responsive family protein was more strongly induced in LA4024. Its homologue in *Arabidopsis* and peach was also found to be regulated by cold stress [60]. The expression of SGN-U215106 was up-regulated by 2.25- and 2.78-fold in LA1777 and LA3969, respectively, whereas only by 0.95-fold in LA4024 under cold stress (Figure S1). SGN-U215106 encodes an auxin-binding F-box protein similar to AtSKP2A. Overexpression of *AtSKP2A* increased tolerance to osmotic stress in *Arabidopsis* [61]. This gene was more strongly induced by cold stress in the two tolerant genotypes, suggesting its up-regulation may confer cold tolerance in tomato.

Five genes involved in the JA biosynthesis pathway were differentially expressed between tolerant and sensitive genotypes under cold stress (Table S9). Among these, two lipoxygenase genes (SGN-U214851 and SGN-U234711) and two 12-oxophytodienoate reductase genes (AJ242551 and SGN-U228308) were more severely repressed in LA4024. However, SGN-U217795 encoding an allene oxide synthase was more strongly induced in LA4024 as compared to LA1777 and LA3969. JA is essential for regulating the systemic defense response in tomato [62]. The depression of JA biosynthesis would affect the expression of JA-responsive genes. Previous studies indicated that the expression of leucine aminopeptidase A1 (*LAP-A1*), *JA2*, pathogenesis-related proteins (*PRs*), and phenylalanine ammonia lyase (*PAL*) were induced by JA [38], [63], [64]. Interestingly, we found *JA2* (SGN-U216055), *PR* (SGN-U215661), *PAL1* (SGN-U239712), and *LAP-A1* (SGN-U212650) were more strongly induced by cold stress in the two tolerant genotypes than in the sensitive one (Figure S1). JA may act as a positive regulator in response to cold stress in tomato.

Both microarray and qPCR analysis showed significantly lower expression of SGN-U223622 in the two tolerant genotypes than in the sensitive one (Table S9). SGN-U223622 encodes a CK oxidase/dehydrogenase, which catalyzes the degradation of CK. The expression of *CKXs* was much lower in the *Arabidopsis* CK-deficient *ipt* mutant and the mutant was more tolerant to salt and drought stress than the wild type [65]. Therefore, SGN-U223622 possibly plays a negative role in response to cold stress in tomato.

### Differentially Expressed Genes Involved in Calcium Signaling

GO term enrichment analysis revealed that the biological process ‘calcium-mediated signaling’ was significantly (p<0.05, FDR as the cut-off) enriched among the differentially expressed genes between tolerant and sensitive genotypes under cold stress (Table S6). A total of 30, 24, and 29 genes involved in calcium regulation were affected by cold stress in LA1777, LA3969, and LA4024, respectively (Table S10). Among them, seven genes were differentially expressed between tolerant and sensitive genotypes. Of these, SGN-U214767, SGN-U220721, SGN-U227216, and SGN-U215654 were more strongly induced by cold stress in the two tolerant genotypes than in the sensitive one (Figure S1; Table S10). The qPCR analysis showed that the expression of SGN-U227216, a gene encoding calcium-binding protein, was significantly higher in the two tolerant genotypes than in the sensitive one under cold stress (p<0.01; up-regulated by 7.16-, 5.17-, 2.41-fold in LA1777, LA3969, and LA4024, respectively). SGN-U214767 and SGN-U220721 encode calcium-binding proteins which are similar to *Arabidopsis* TCH2 and AtCP1, respectively. Previous studies indicated that *TCH2* and *AtCP1* were induced by cold stress in *Arabidopsis* [66], [67]. SGN-U215654 encodes a putative calmodulin. Calcium-binding proteins and calmodulin are Ca^2+^ sensors that sense changes in cellular Ca^2+^ and regulate the expression of downstream genes [68]. The up-regulation of these four Ca^2+^ sensors was significantly higher in the two tolerant genotypes under cold stress, suggesting these might act as positive regulators in tolerance to cold stress in tomato. Three genes were more strongly induced in the sensitive genotype. Both SGN-U221694 and SGN-U231357 encode sodium/calcium exchanger family proteins, and SGN-U214886 encodes a calmodulin-dependent protein kinase, similar to NtCPK4. In tobacco, *NtCPK4* expression was induced by salt stress and gibberellin [69].

**Table 2:** Significantly altered biochemical pathways and their corresponding gene expression between tolerant and sensitive tomato genotypes under cold stress.

Unigene ID	Relative expression level^A^			Annotation
	LA1777	LA3969	LA4024	
**Jasmonic acid biosynthesis**				
SGN-U234711	0.81	−1.16	−2.34	Lipoxygenase
SGN-U214851	1.02	−1.22	−2.36	Lipoxygenase
AJ242551	−1.08	−0.91	−2.41	12-oxophytodienoate reductase
SGN-U228308	−0.69	−0.81	−2.63	12-oxophytodienoate reductase
SGN-U217795	3.27	3.45	4.45	Allene oxide synthase
**Brassinosteroid metabolic process**				
SGN-U221699	0.67	1.77	2	Cytochrome P450
SGN-U233201	5.39	3.73	2.59	UDP-glucosyltransferase
SGN-U215317	5.52	5.63	3.95	UDP-glucosyltransferase
SGN-U221349	6.11	4.27	3.1	UDP-glucosyltransferase
**Starch degradation**				
SGN-U213712	−0.56	−1.7	−2.24	Beta-amylase
**Phenylpropanoid biosynthesis, initial reactions**				
SGN-U239712	3.04	2.66	1.88	Phenylalanine ammonialyase
**Leucine biosynthesis**				
SGN-U213969	0.38	2.5	3.2	2-isopropylmalate synthase A
**Calvin cycle**				
SGN-U225498	4.24	3.46	2.28	RuBisCO small subunit 3B
**Removal of superoxide radicals**				
SGN-U232054	−4.12	−4	−2.66	Catalase

The 92 genes with significant differences in expression between tolerant and sensitive genotypes at 3 d of cold treatment (4°C) were analyzed for significantly (p<0.05) altered biochemical pathways using the Plant MetGenMAP system [33]. (A) Positive (up-regulated) and negative (down-regulated) expression values (log_2_ ratio cold stress/control) are means of three independent biological replicates.

**Figure 6:**
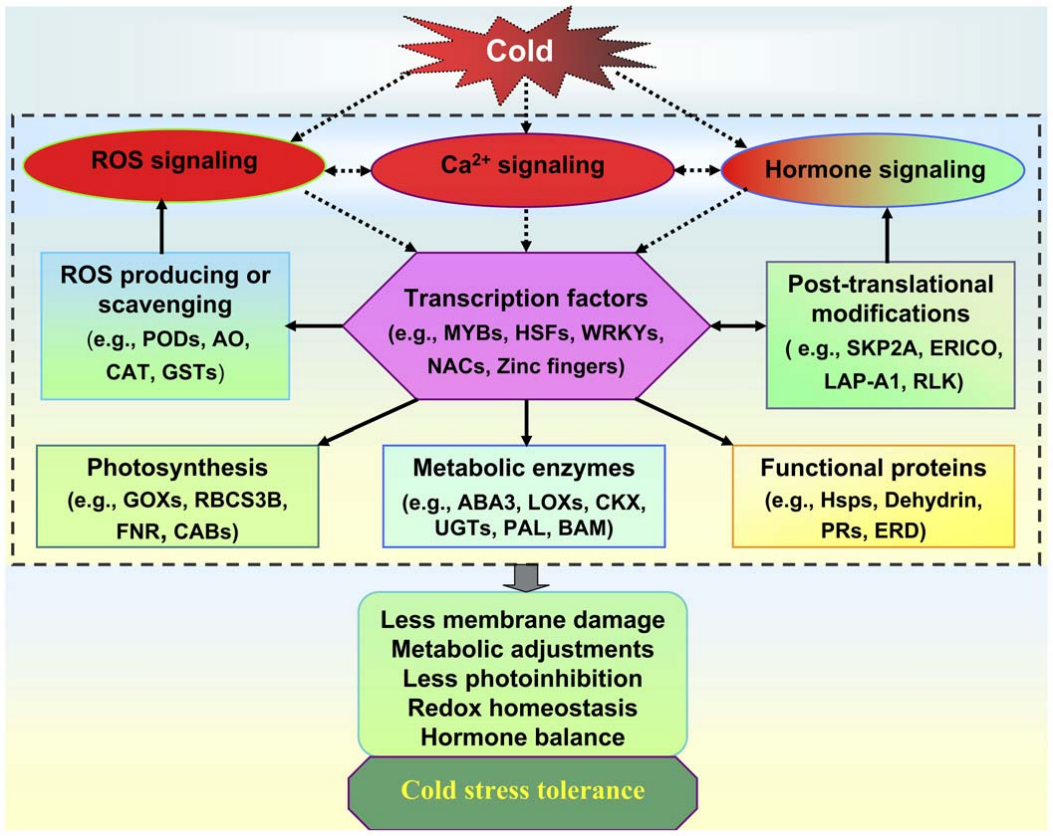
Hypothetical working model of cold tolerance in tomato mediated by the introgressed chromosomal segment of *S. habrochaites* LA1777. ROS, calcium, and hormones as signaling molecules may play critical roles in tomato adaptation to cold stress. The modulation of these signaling pathways then activated transcription regulation. The differential expression of transcription factors then caused many transcripts modified between tolerant and sensitive genotypes, such as functional proteins, post-translational modification proteins, and genes associated with physiological and metabolic processes, such as photosynthesis, ROS production or scavenging system, and metabolic enzymes. The modulation of the expression of genes associated with post-translational modifications, ROS, and hormones then feedback to fine-tune transcription factors, ROS, and hormone signaling networks. These specific modifications make LA3969 be close to its donor parent LA1777, and both showed more cold tolerant than the recurrent parent LA4024. ROS, reactive oxygen species; *AO,* amine oxidase; *PODs, peroxidases;* GSTs, glutathione S-transferases; CAT, catalase; RBCS3B, rubisco small subunit 3B; FNR, ferredoxin-NADP^+^-oxidoreductase; CABs, chlorophyll a/b binding proteins; GOXs, glycolate oxidases; LOXs, lipoxygenases; ABA3, ABA deficient 3; UGTs, UDP-glucosyltransferases; CKX, cytokinin oxidase/dehydrogenase; PAL, phenylalanine ammonia-lyase; PRs, pathogenesis-related proteins; BAM, beta-amylase; Hsps, heat shock proteins; ERD, early response to dehydration; HSFs, heat shock factors; SKP2A, S-phase kinase-associated protein 2A; RLK, receptor-like protein kinase; LAP-A1, leucine aminopeptidase A1.

### Differentially Expressed Transcription Factors

Transcription factors (TFs) play crucial roles in regulating the expression of stress-responsive genes under biotic and abiotic stresses. A larger number of TFs were regulated by cold stress at least in one tomato genotype. Representative ZF families included MYB, NAC, WRKY, AP2/EREBP, HSF (heat shock factor), bHLH, bZIP, and zinc finger (Figure S4). Among these, ten ZFs were specifically expressed in the two tolerant genotypes under cold stress (Figure S4A). Sixteen TFs were regulated by cold stress exclusively in LA4024, and most of them were down-regulated (Figure S4B). A total of 96 TFs were regulated by cold stress common to all three genotypes. Nearly half of them belong to NAC, WRKY, and zinc finger ZF superfamilies (Figure S4C). NAC, WRKY and zinc finger TFs play important roles in response to abiotic stress by controlling stress-responsive gene expression and/or modifying other signaling and regulatory networks [70]–[72]. So many NAC, WRKY, and zinc finger TFs were induced in all three genotypes, depicting the development of complex and highly interconnected regulatory networks in response to cold stress in tomato. All cold-responsive genes, belonging to NAC, WRKY, AP2/EREBP, and HSF gene families, were enhanced in all three genotypes. The up-regulation of two *WRKYs* (SGN-U215123 and SGN-U213637) was confirmed by qPCR (Table S3). Many tomato WRKY TFs were also found to be induced by salt or drought stress [20], [21]. Therefore, the up-regulation of WRKY TFs may be a general stress response common to various abiotic stresses in tomato.

Among these cold-responsive TFs, eight showed significant changes in transcript abundance between tolerant and sensitive genotypes under cold stress, including SGN-U221957, SGN-U224924, SGN-U218469, SGN-U234004, SGN-U215123, SGN-U216055, SGN-U216769, and SGN-U215577 (Figure S4). SGN-U221957 and SGN-U224924 encode HSFs. The expression of SGN-U221957 was down-regulated by 2.58-fold in LA4024, whereas it was slightly up-regulated in LA1777 and LA3969 (0.22- and 0.72-fold, respectively) under cold stress. SGN-U224924 was more strongly up-regulated by cold stress in the two tolerant genotypes than the sensitive one (Figure S1). HSFs play a central role in regulating the expression of heat shock proteins (*Hsps*) in response to heat and other stress stimuli [73]. We found that the expression of two *Hsps* (SGN-U212639 and SGN-U212643) was significantly higher in the two tolerant genotypes than in the sensitive one under cold stress (Figure S1). SGN-U216769 (*SAP11*) encoding an A20/AN1 zinc finger protein that has been found to be induced by cold stress in tomato [47]. SGN-U234004 and SGN-U218469 encode MYBs similar to *Arabidopsis* MYB15 and MYB107. *MYB15* is a negative regulator of cold tolerance in *Arabidopsis* [42]. These two *MYBs* were more strongly induced in the sensitive genotype adumbrating them as negative regulators of cold tolerance in tomato. SGN-U215577, encoding a homologue of *Arabidopsis* ASPG1 (aspartic protease in guard cell 1), was more severely suppressed by cold stress in LA4024. Overexpression of the *ASPG1* gene conferred drought avoidance in *Arabidopsis* by up-regulating the expression of drought- and/or ABA-inducible genes, such as *KIN1*, *KIN2*, *RAB18*, and *RD26* [74]. *KIN1*, *KIN2*, and *RAB18* are also cold-inducible genes [75]–[77]. These results suggest that the *ASPG1* gene may also play a positive role in plant adaptation to cold stress by activating the downstream stress-responsive genes.

### Differentially Expressed Genes Involved in Post-translational Modifications

Post-translational modifications play important roles in rapid and fine-tuned regulation of transcription factors under abiotic stress [78]. A total of 171 genes associated with post-translational modifications were regulated by cold stress in at least one tomato genotype (Table S11). Among these, six showed significant differences in expression between tolerant and sensitive genotypes under cold stress, including SGN-U215679, SGN-U212650, SGN-U229977, SGN-U234375, SGN-U220612, and SGN-U215106 (Figure S1; Table S11). SGN-U220612 and SGN-U234375 encoding RING-H2-type zinc-finger proteins similar to *Arabidopsis* XERICO were more strongly induced by cold stress in the two tolerant genotypes than in the sensitive one (Table S11). Overexpression of *XERICO* enhances drought tolerance by accumulating more ABA in *Arabidopsis* [79]. The significantly higher up-regulation of these two unigenes in the two tolerant genotypes suggested that they might act as positive regulators in the cold stress response in tomato. Another gene, SGN-U215106, encodes a homologue of *Arabidopsis* SKP2A. SKP2A is an auxin-binding F-box protein, and its overexpression in transgenic *Arabidopsis* confers tolerance to osmotic stress [61]. F-box proteins are part of the ubiquitin ligase SCF complex that catalyzes the degradation of the Aux/IAA proteins through ubiquitination pathway [80]. Previous studies indicated that auxin regulates transcription by promoting the degradation of the Aux/IAA proteins [81]. The remarkable increase in the expression of this gene in the two tolerant genotypes may activate the auxin-mediated transcription under cold stress. Interestingly, we found the GO term ‘response to auxin’ was significantly enriched among the differentially expressed genes between tolerant and sensitive genotypes under cold stress. These results suggested a potential link between cold and auxin signaling pathways.

### Biochemical Pathways Significantly Altered Between Tolerant and Sensitive Genotypes under Cold Stress

To identify the significantly altered biochemical pathways between cold-tolerant and -sensitive tomato genotypes under cold stress, the 92 differentially expressed genes were analyzed using the Plant MetGenMap system [33]. Seven biochemical pathways varied significantly between tolerant and sensitive genotypes under cold stress, including jasmonic acid biosynthesis, brassinosteroid metabolic process, phenylpropanoid biosynthesis, starch degradation, leucine biosynthesis, Calvin cycle, and removal of superoxide radicals (Table 2). The phenylpropanoid biosynthesis and Calvin cycle pathways were significantly enhanced in the two tolerant genotypes under cold stress compared with the sensitive one. Three UDP-glucosyltransferase genes involved in brassinosteroid metabolic process were more strongly induced by cold stress in the two tolerant genotypes than in the sensitive one. Some genes from UDP-glucosyltransferase family genes have been found to be regulated by abiotic and/or biotic stresses in *Arabidopsis* [82]. SGN-U213712, encoding a beta-amylase involved in starch degradation, was more severely suppressed in the sensitive genotype. Previous studies indicate that the increase in beta-amylase activity is correlated with maltose accumulation which contributes to the protection of the PSII photochemical efficiency, proteins, and membranes during freezing stress [83], [84]. Therefore, the significant repression of this beta-amylase gene may decrease maltose content and reduce PSII photochemical efficiency in the cold-sensitive genotype under cold stress. In agreement with this, we found that the PSII photochemical efficiency was repressed more severely in LA4024 after 7 d of cold stress treatment (Figure 5C).

### Conclusions

In this study, a total of 93 *S. habrochaites* LA1777 ILs, together with their donor (*S. habrochaites* LA1777) and recurrent (*S. lycopersicum* LA4024) parent were evaluated for cold tolerance at the seedling stage. The IL LA3969 and its donor parent were found to be more cold tolerant than the recurrent parent during cold stress (Figures 1A to 1E). To better understand the mechanisms of cold tolerance in tomato, differences in stress-related physiological indicators and global gene expression between cold-tolerant (LA3969 and LA1777) and -sensitive (LA4024) genotypes under cold stress were investigated. During cold stress, the two tolerant genotypes showed less severe membrane damage, less photoinhibition of PSII, and lower APX activities than the sensitive genotype. Comparative transcriptome analysis revealed 92 genes were differentially expressed between tolerant and sensitive genotypes after 3 d of cold stress (4°C). A total of 80 genes with significant differences in expression between *S. habrochaites* and *S. lycopersicum* were mapped to the introgressed chromosomal regions of the 22 selected cold-tolerant ILs and/or cold tolerance QTLs reported previously in *S. habrochaites* [7], [12], [13]. According to chromosomal location, expression differences, and gene annotation, 30 genes that are most likely to confer cold tolerance in *S. habrochaites* were identified (Table 1).

ROS, calcium, and hormones as signaling molecules may play critical roles in tomato adaptation to cold stress. The modulation of these signaling pathways could activate directly stress response-related genes or interact directly/indirectly with several other signaling networks to regulate transcription. The activated TFs (e.g., MYBs, HSFs, WRKYs, NACs, and zinc fingers) then caused differential expression of many transcripts among tolerant and sensitive genotypes, such as functional proteins (e.g., HSPs, PRs, and dehydrin), post-translational modification proteins (e.g., SKP2A, LAP-A1, and XERICOs), and genes associated with physiological and metabolic processes, such as photosynthesis (e.g., FNR, GOXs, and RBCS3B), ROS production or scavenging system (e.g., PODs, AO, and GSTs), hormone biosynthesis and metabolism (ABA3, LOXs, and UGTs), and other metabolic enzymes (e.g., PAL and BAM). These specific modifications make LA1777 and LA3969 more cold tolerant than LA4024, by reducing cell membrane damage and photoinhibition, regulating metabolism, and maintaining hormone and ROS homeostasis. Based on our results, we summarized a hypothetical working model for the potential roles of this introgressed chromosomal segment from *S. habrochaites* LA1777 in the regulation of cold stress tolerance (Figure 6).

To the best of our knowledge, this is the first report comparing the differences in global gene expression between tolerant and sensitive genotypes under cold stress in tomato. The results not only provide new insights into the molecular mechanisms of cold tolerance in tomato, but also provide potential candidate genes for genetic improvement of the cultivated tomato.

## Supporting Information

Figure S1 **Heat map of genes significant differentially expressed between tolerant and sensitive tomato genotypes under cold stress.** Cold-responsive genes with statistically significant differences (p<0.05, Student’s t test) in expression between tolerant and sensitive genotypes were clustered using Genesis software [35]. The color intensity represents the gene expression value (log_2_ ratio cold stress/control), as indicated by the color scale. The corresponding gene expression values obtained from the microarray results are also shown. Genes shown in red indicate they are mapped to the introgressed chromosomal segments of the 22 selected cold-tolerant ILs and/or cold tolerance QTLs identified previously in *S. habrochaites* [7], [12], [13]. Chr, chromosomal localization of genes.
(TIF)

Figure S2 **Correlation analysis of gene expression values obtained from microarray and qPCR analysis.** The expression ratio (log_2_ ratio stress/control) is presented as mean of three replicates. The Pearson correlation coefficient (*r*) is indicated in the figure.
(TIF)

Figure S3 **Comparative analysis of ROS-related gene expression, enzymatic activity, and ROS accumulation among the three tomato genotypes under cold stress.** (A) Relative expression levels of selected ROS-related genes under cold stress. Leaf samples from control and cold-treated (3 d at 4°C) plants were used for qPCR analysis. *EF1α* expression was used as internal control. The relative expression level is shown as log_2_ ratio (cold stress/control). (B to E) Changes in activities of APX, *POD*, CAT, and SOD in the leaves of the three tomato genotypes treated at 4°C for 0, 1, 3, 5, and 7 d. Data are presented as mean ± SE of three independent biological replicates. Asterisks indicate a significant difference between the tolerant and sensitive genotypes based on Student’s t test. *, p<0.05; **, p<0.01. (F, G) Histochemical staining of H_2_O_2_ and O_2_^-^ accumulation in the leaves of the three tomato genotypes treated at 4°C for 0, 1, 3 d. Six-week-old seedlings were treated at 4°C for the indicated time points. Plants grown at 25°C were used as control. DAB and NBT stains were used to detect H_2_O_2_ and O_2_^-^, respectively. The brown and dark blue regions on the leaves indicate the generation of H_2_O_2_ and O_2_^-^, respectively. The samples shown are representative of six replicates.
(TIF)

Figure S4 **Heat map representation of transcription factor expression in the three tomato genotypes under cold stress.** Cold-responsive transcription factors identified in both tolerant genotypes (A), exclusively in the sensitive genotype (B), and common to all three genotypes (C). The expression images were generated using Genesis software [35]. The color intensity represents the gene expression value (Log_2_ ratio stress/control), as indicated by the color scale. The corresponding gene expression values obtained from microarray results are also shown. Asterisk indicates a significant difference in gene expression between tolerant and sensitive genotypes (p<0.05, Student’s t test).
(TIF)

Table S1 **List of primer sequences used for qPCR analysis.**
(DOC)

Table S2 **List of genes whose expression level in LA777 is significantly (p<0.01) different from that in LA3969 and LA4024 under cold stress.**
(XLS)

Table S3 **Confirmation of microarray data by qPCR.**
(DOC)

Table S4 **Relative expression levels of previously reported cold-responsive genes in the microarray results.**
(DOC)

Table S5 **Significantly enriched GO biological process terms among the up- and down-regulated genes in the three tomato genotypes under cold stress.**
(XLS)

Table S6 **Significantly enriched GO biological process terms among the differentially expressed genes between tolerant and sensitive tomato genotypes under cold stress.**
(DOC)

Table S7 **List of cold-responsive genes involved in photosynthesis in the three tomato genotypes.**
(XLS)

Table S8 **List of cold-responsive genes involved in ROS generation and scavenging in the three tomato genotypes.**
(XLS)

Table S9 **List of cold-responsive genes involved in hormone metabolism and signaling pathways in the three tomato genotypes.**
(XLS)

Table S10 **List of cold-responsive genes involved in calcium regulation in the three tomato genotypes.**
(XLS)

Table S11 **List of cold-responsive genes involved in post-translational modifications in the three tomato genotypes.**
(XLS)
