# Supplementary figures and images for: Differential Modulation of Photosynthesis, Signaling, and Transcriptional Regulation between Tolerant and Sensitive Tomato Genotypes under Cold Stress
Source: PLoS One. 2012 Nov 30;7(11):e50785. doi: 10.1371/journal.pone.0050785 (PMC3511270; doi:10.1371/journal.pone.0050785)

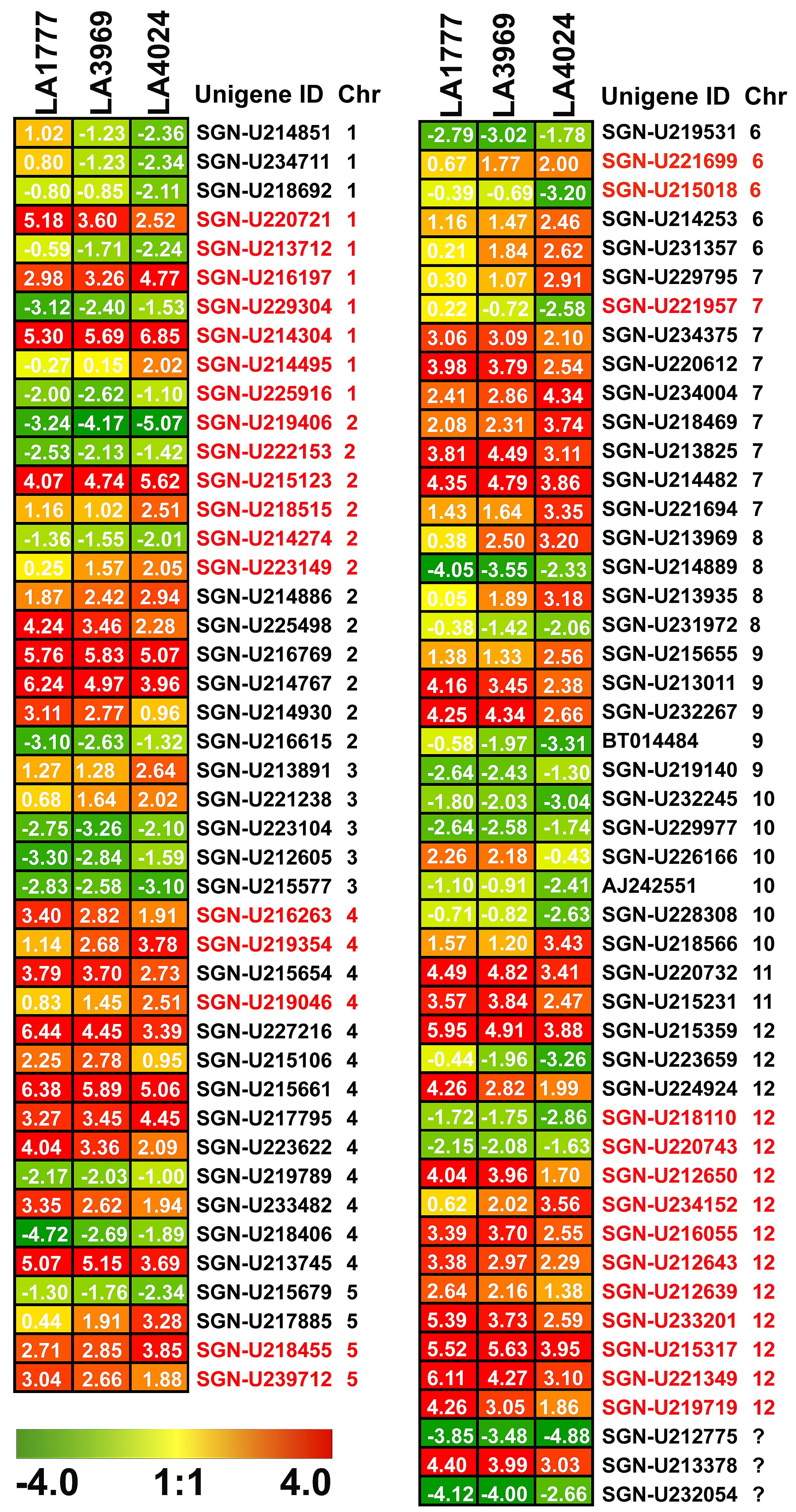

Supplement: Figure S1 — Heat map of genes significant differentially expressed between tolerant and sensitive tomato genotypes under cold stress. Cold-responsive genes with statistically significant differences (p<0.05, Student’s t test) in expression between tolerant and sensitive genotypes were clustered using Genesis software [35]. The color intensity represents the gene expression value (log2 ratio cold stress/control), as indicated by the color scale. The corresponding gene expression values obtained from the microarray results are also shown. Genes shown in red indicate they are mapped to the introgressed chromosomal segments of the 22 selected cold-tolerant ILs and/or cold tolerance QTLs identified previously in S. habrochaites [7], [12], [13]. Chr, chromosomal localization of genes. (TIF) [file pone.0050785.s001.tif]

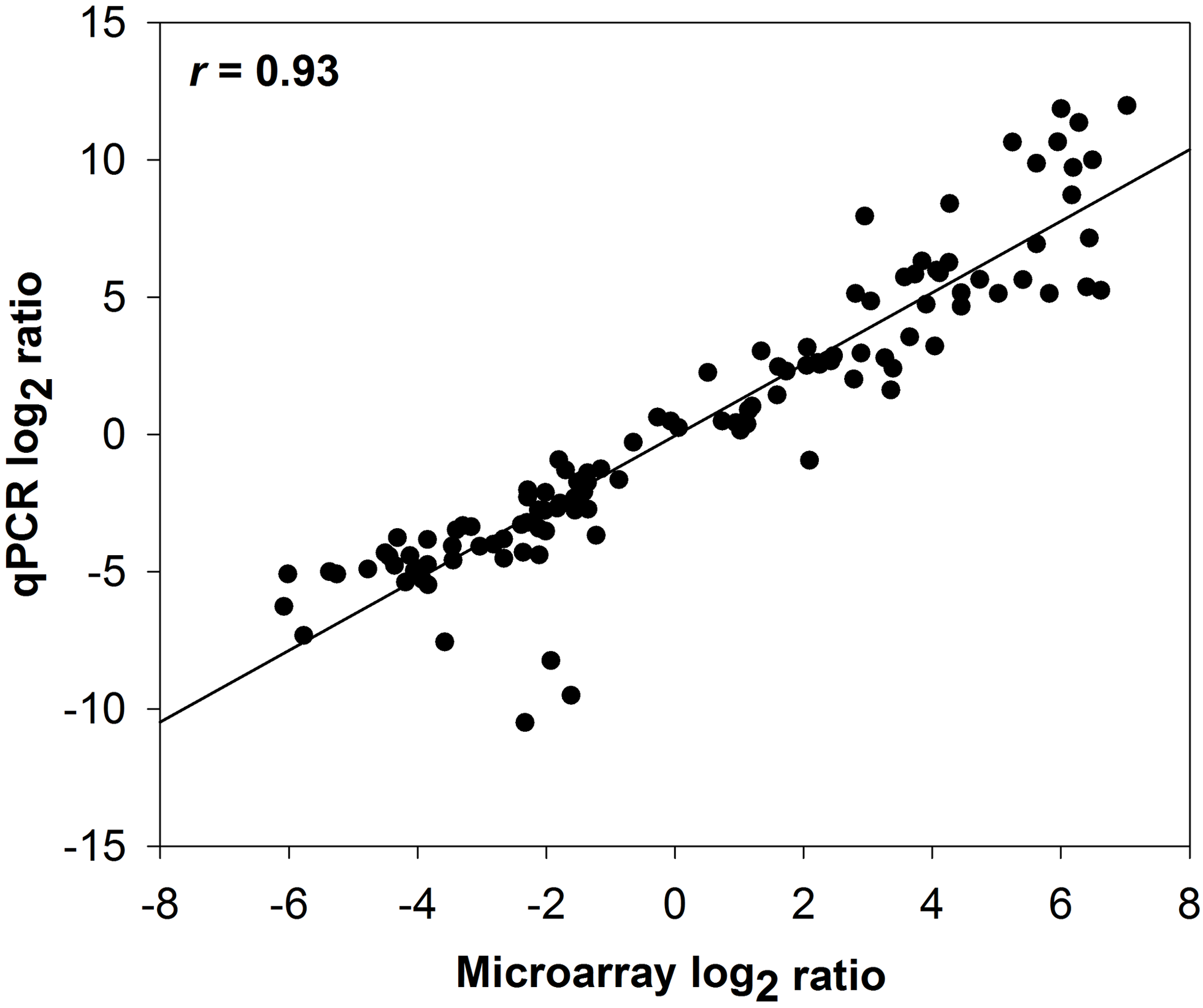

Supplement: Figure S2 — Correlation analysis of gene expression values obtained from microarray and qPCR analysis. The expression ratio (log2 ratio stress/control) is presented as mean of three replicates. The Pearson correlation coefficient (r) is indicated in the figure. (TIF) [file pone.0050785.s002.tif]

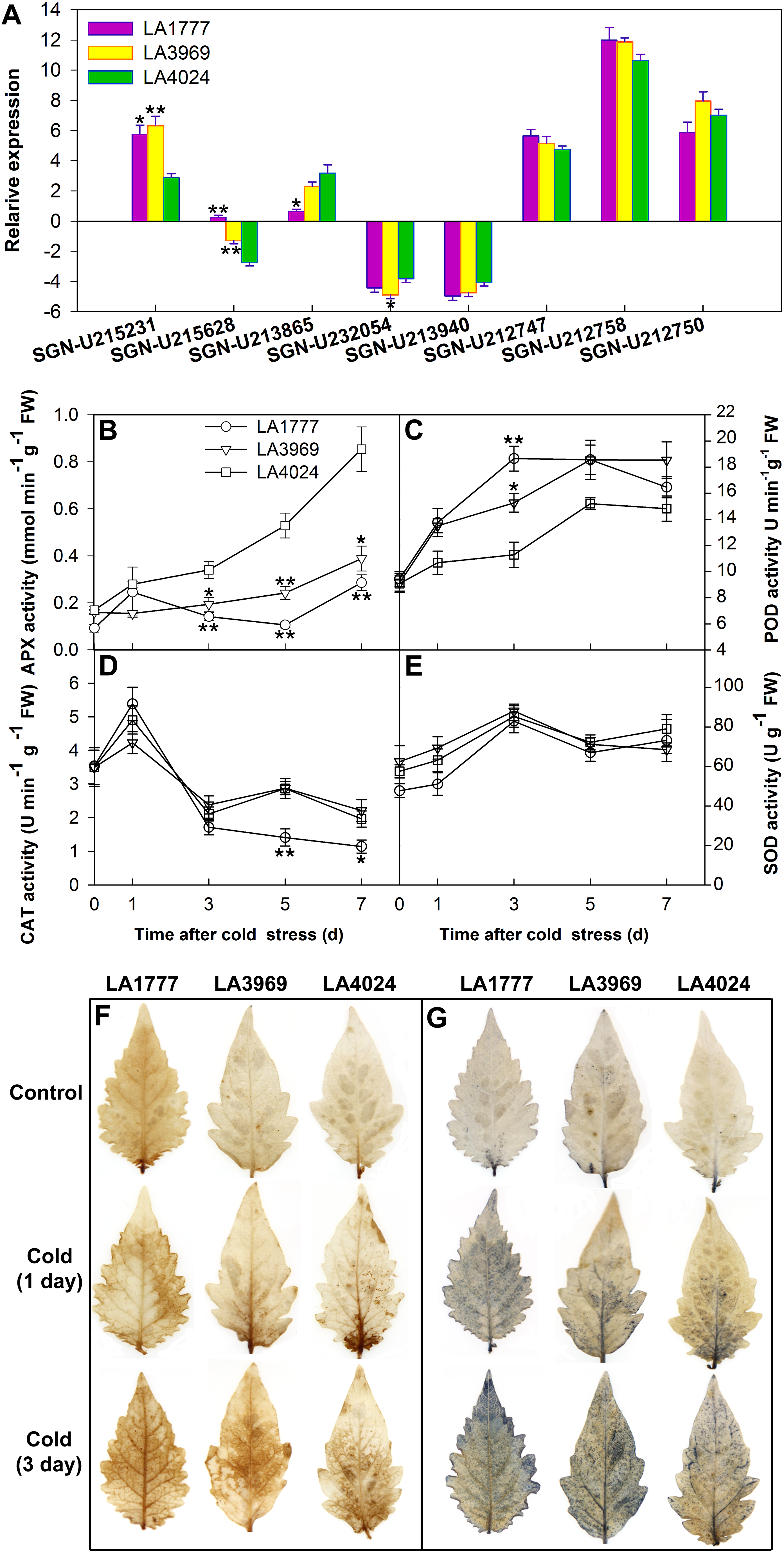

Supplement: Figure S3 — Comparative analysis of ROS-related gene expression, enzymatic activity, and ROS accumulation among the three tomato genotypes under cold stress. (A) Relative expression levels of selected ROS-related genes under cold stress. Leaf samples from control and cold-treated (3 d at 4°C) plants were used for qPCR analysis. EF1α expression was used as internal control. The relative expression level is shown as log2 ratio (cold stress/control). (B to E) Changes in activities of APX, POD, CAT, and SOD in the leaves of the three tomato genotypes treated at 4°C for 0, 1, 3, 5, and 7 d. Data are presented as mean ± SE of three independent biological replicates. Asterisks indicate a significant difference between the tolerant and sensitive genotypes based on Student’s t test. *, p<0.05; **, p<0.01. (F, G) Histochemical staining of H2O2 and O2 - accumulation in the leaves of the three tomato genotypes treated at 4°C for 0, 1, 3 d. Six-week-old seedlings were treated at 4°C for the indicated time points. Plants grown at 25°C were used as control. DAB and NBT stains were used to detect H2O2 and O2 -, respectively. The brown and dark blue regions on the leaves indicate the generation of H2O2 and O2 -, respectively. The samples shown are representative of six replicates. (TIF) [file pone.0050785.s003.tif]

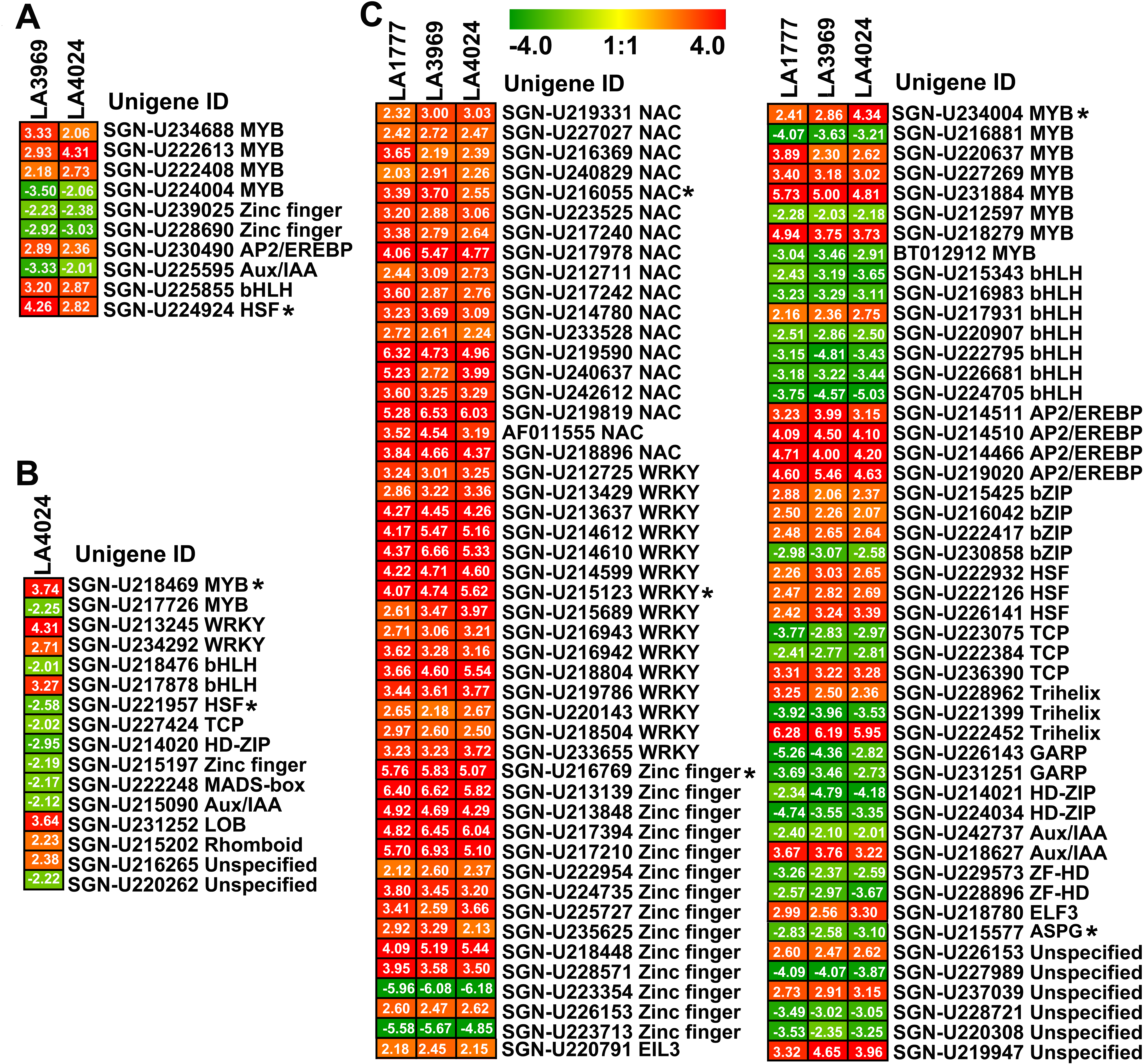

Supplement: Figure S4 — Heat map representation of transcription factor expression in the three tomato genotypes under cold stress. Cold-responsive transcription factors identified in both tolerant genotypes (A), exclusively in the sensitive genotype (B), and common to all three genotypes (C). The expression images were generated using Genesis software [35]. The color intensity represents the gene expression value (Log2 ratio stress/control), as indicated by the color scale. The corresponding gene expression values obtained from microarray results are also shown. Asterisk indicates a significant difference in gene expression between tolerant and sensitive genotypes (p<0.05, Student’s t test). (TIF) [file pone.0050785.s004.tif]
