# Supplementary material for: Differential Modulation of Photosynthesis, Signaling, and Transcriptional Regulation between Tolerant and Sensitive Tomato Genotypes under Cold Stress
Source: PLoS One. 2012 Nov 30;7(11):e50785. doi: 10.1371/journal.pone.0050785 (PMC3511270; doi:10.1371/journal.pone.0050785)
Supplement: Table S1 — List of primer sequences used for qPCR analysis. (DOC) [file pone.0050785.s005.doc]

**Table S1. List of primer sequences used for qPCR analysis.**

| **Unigene ID** | **Forward primer (5’-3’)** | **Reverse primer (5’-3’)** |
| --- | --- | --- |
| X14449 (EF1a) | CGTGGTTATGTTGCCTCAAA | ACAGCAATGTGGGAAGTGTG |
| SGN-U213019 | TTCACGTATTGTGGCTGCTT | CCAGTCCCATTAGATTATCGTTG |
| SGN-U213139 | AATGGGAGGGTTTGTTGTTG | TCGACTTCGTCAACACTGGT |
| SGN-U213637 | GATTTGGTGGATGATGGTCA | TGGGTTGTCTTGGATTTTCTG |
| SGN-U214067 | CAAGACTGAGAGCCGATTCC | CATCCCCAACTAATGCGACT |
| SGN-U214167 | GGGTTCATTCCTTGGTGAAA | TTAAGCCACGATACGCTCCT |
| SGN-U215106 | GGTCCCTCGGCCTATACTTC | CCTCGTTCCTGTGCTTCTTC |
| SGN-U215123 | GAAGCAACCATGAGGAAAGC | GTGGACATGGATTGCCTTTT |
| SGN-U215389 | CCCTGGAAGGAATGTGATGT | CCCAAAGGATGAGAGCAAAG |
| SGN-U216350 | CAATTGCCCTAACGTTGTTG | AGCATCAGTAGCAGCAGCAG |
| SGN-U222452 | GAGGCAGAAGGTGGTGTAGG | TGCCAACTTTTTCTCCATCA |
| SGN-U227216 | CACGAGGATCGAGAAAATGTT | AATGCTCTTTGCAGCTCCTC |
| SGN-U228673 | TCAAACCCTTGCTTGAGAATC | TGCTTTGTTCCAACTCATCG |
| SGN-U233360 | TTCCGTCATTCACCGTTTC | TCATCCTCGAGAACAAAGCA |
| SGN-U218904 | GGCCCTGACCGTGTTAAGTA | CTGAAAGTCCAGCGGTATCC |
| SGN-U218911 | CCAAGCCTGTCTCTTCTGGT | GTGTCCCATCCGTAGTCACC |
| SGN-U232496 | AATGTGGGAAGGGTGAAGTG | AAATGGAAGCCCAGTTCCTT |
| SGN-U231963 | GGGCTATGGCAAACGTAAGA | TGAGTCTGGGCAGCAACATA |
| SGN-U232245 | TCAATGGACTTTGGCTGAGA | GCATAGCTGTTGGTGCAATC |
| SGN-U213321 | ATGCAGTGATGTTTCCACCA | GCTGTATCCCCACTCGTTGT |
| SGN-U232616 | ACAGACGAAGCACCCTGAAT | GGCAAATAAATCTGACGCATAA |
| SGN-U 215231 | CTTGCCCTAATGCTCTCACC | GCATCACAACCCTGAACAAA |
| SGN-U215628 | CATCACAGTTTGGCTTGCTG | TTCGACCAGGAATGGCTATC |
| SGN-U213865 | TTCTTGTTGATGCCCAGACA | CAAACTGCTCCTTGGTTGCT |
| SGN-U232054 | GTGCATTGAAACCAAATCCA | AATCCGTTGGGAGACAAACA |
| SGN-U213940 | TTTCTGGGAAGCAAATCGTC | TTGGGCAATGAAGAAGAAGC |
| SGN-U212747 | GCAAGCCCATTTGTGAGTCT | TGCTGACCCCTTATCATCG |
| SGN-U212758 | GGAGCAAAAGAGAGGAAAAAGA | GCAGCAACATCAACAAATCC |
| SGN-U212750 | TGGAGAGATGAGTACGTTAATTGC | GCAGCTTGAGCACGAGTGA |
| SGN-U214274 | GGGTGTCAAACTATGCTCTCG | TTTCTTATTCGGACGCAAGG |
| SGN-U227354 | GGTTTGGGATGTGATGTCAA | TTTCCACGCACAAATAGCAC |
| SGN-U229261 | CCCTGCATCTGACGGTTTAT | CAGAACCTCGTGAAACAGCA |
| SGN-U221937 | ATTGTCGTGCTGTTGTGAGC | CCCGATGCCTCAATTCTATC |
| SGN-U215101 | TTGGTGCTATTTCGGGTAGTG | TGGACAACCTTTCTGCAACC |
| SGN-U223813 | ACCTCCATTACCGTCCAACA | CCTACACCACCGTTCGATCT |
| SGN-U226246 | CTTCAGAGAGGGCATTTGGA | GGCAGTGTGTGCGTAACCTA |
| SGN-U214851 | TCATTTTCCCCTGGCAAGTA | TGGTGCATTTGGATCTTCCT |
| SGN-U239057 | ATCCACGCTTTTCCAATCAG | TAGCCATGGATGAGGGACAT |
| SGN-U223622 | ATTCCAGCTTCGGAGTTCAC | TGGCACTTCCCATAAACCTT |
| SGN-U216135 | GGACATTCCATTAGGGGACA | CCCAAGAAACCCTAACACCA |
| SGN-U215482 | GGCTTTAGATCAGCCCAATG | TGTTCTTCGCAGCAGTTACG |
| SGN-U226143 | TCCGTAAGCAGTGGTGATGA | CGAACCTACATCCGAAGCTG |
