# Supplementary material for: Differential Modulation of Photosynthesis, Signaling, and Transcriptional Regulation between Tolerant and Sensitive Tomato Genotypes under Cold Stress
Source: PLoS One. 2012 Nov 30;7(11):e50785. doi: 10.1371/journal.pone.0050785 (PMC3511270; doi:10.1371/journal.pone.0050785)
Supplement: Table S3 — Confirmation of microarray data by qPCR. (DOC) [file pone.0050785.s007.doc]

**Table S3. Confirmation of microarray data by qPCR.** Relative expression levels of selected genes in the three genotypes at 3 d of cold stress (4 °C). Values are shown as log_2_ ratio stress/control. Data are means of three independent biological replicates. ‘--’ represent the missing values.

| Uingene ID | LA1777 | | LA3969 | | LA4024 | |
| --- | --- | --- | --- | --- | --- | --- |
|  | Microarry | qRT-PCR | Microarry | qRT-PCR | Microarry | qRT-PCR |
| SGN-U227216 | 6.44 | 7.16 | 4.45 | 5.17 | 3.39 | 2.41 |
| SGN-U213139 | 6.40 | 5.38 | 6.62 | 5.25 | 5.82 | 5.14 |
| SGN-U222452 | 6.28 | 11.37 | 6.19 | 9.73 | 5.95 | 10.66 |
| SGN-U216350 | 6.17 | 8.73 | 6.49 | 10.00 | 5.62 | 9.88 |
| SGN-U213637 | 4.27 | 8.41 | 4.45 | 4.67 | 4.26 | 6.27 |
| SGN-U215123 | 4.07 | 5.99 | 4.74 | 5.65 | 5.62 | 6.95 |
| SGN-U215106 | 2.25 | 2.56 | 2.78 | 2.02 | 0.95 | 0.43 |
| SGN-U213019 | 1.59 | 1.44 | 1.34 | 3.04 | -1.36 | -1.40 |
| SGN-U214167 | 0.73 | 0.49 | 1.15 | 0.89 | 1.14 | 0.90 |
| SGN-U228673 | -0.65 | -0.28 | -0.07 | 0.49 | 0.51 | 2.26 |
| SGN-U215389 | -1.81 | -0.92 | -1.56 | -2.30 | 1.12 | 0.39 |
| SGN-U233360 | -2.66 | -4.52 | -3.30 | -3.33 | -3.40 | -3.48 |
| SGN-U214067 | -5.77 | -7.33 | -3.84 | -5.48 | -3.93 | -5.28 |
| SGN-U218904 | -4.51 | -4.32 | -4.19 | -5.39 | -6.08 | -6.27 |
| SGN-U218911 | -2.39 | -3.29 | -4.78 | -4.91 | -5.37 | -5.00 |
| SGN-U232496 | -0.87 | -1.64 | -1.83 | -2.68 | -2.12 | -3.43 |
| SGN-U231963 | -1.79 | -2.50 | -1.35 | -2.72 | -2.11 | -4.40 |
| SGN-U232245 | -1.52 | -1.73 | -2.03 | -2.76 | -3.04 | -4.08 |
| SGN-U213321 | -4.31 | -3.77 | -4.44 | -4.44 | -6.02 | -5.09 |
| SGN-U232616 | -1.93 | -8.24 | -1.62 | -9.51 | -2.33 | -10.50 |
| SGN-U215231 | 3.57 | 5.74 | 3.84 | 6.32 | 2.47 | 2.87 |
| SGN-U215628 | 0.05 | 0.26 | -1.70 | -1.29 | -2.12 | -2.75 |
| SGN-U213865 | -0.27 | 0.63 | 1.73 | 2.31 | 2.05 | 3.17 |
| SGN-U232054 | -4.12 | -4.42 | -4.00 | -4.89 | -2.66 | -3.81 |
| SGN-U213940 | -4.05 | -4.96 | -3.85 | -4.74 | -3.46 | -4.07 |
| SGN-U212747 | 5.41 | 5.64 | 5.03 | 5.14 | 3.90 | 4.75 |
| SGN-U212758 | 7.03 | 11.99 | 6.00 | 11.87 | 5.25 | 10.65 |
| SGN-U212750 | 4.11 | 5.89 | 2.95 | 7.95 | -- | 7.01 |
| SGN-U214274 | -1.36 | -1.75 | -1.55 | -2.76 | -2.01 | -3.53 |
| SGN-U227354 | -2.29 | -2.01 | -3.58 | -7.57 | -- | -7.62 |
| SGN-U229261 | 3.04 | 4.86 | 2.81 | 5.14 | 3.73 | 5.85 |
| SGN-U221937 | 2.05 | 2.52 | 2.43 | 2.68 | 2.89 | 2.97 |
| SGN-U215101 | -- | 2.39 | 1.61 | 2.47 | 2.22 | 2.63 |
| SGN-U223813 | -2.29 | -2.28 | -3.30 | -3.33 | -3.85 | -3.83 |
| SGN-U215106 | 2.25 | 2.56 | 2.78 | 2.02 | 0.95 | 0.43 |
| SGN-U226246 | -1.42 | -2.09 | -- | -3.15 | -2.31 | -3.21 |
| SGN-U214851 | 1.02 | 0.16 | -1.23 | -3.68 | -2.36 | -4.30 |
| SGN-U239057 | -3.45 | -4.59 | -- | -3.82 | -3.17 | -3.37 |
| SGN-U223622 | 2.09 | -0.94 | 3.36 | 1.62 | 4.04 | 3.22 |
| SGN-U216135 | 1.20 | 1.03 | 3.26 | 2.80 | 3.65 | 3.55 |
| SGN-U215482 | -2.02 | -2.11 | -1.45 | -1.67 | -1.15 | -1.26 |
| SGN-U226143 | -5.26 | -5.09 | -4.36 | -4.77 | -2.82 | -4.00 |
