# Supplementary material for: Differential Modulation of Photosynthesis, Signaling, and Transcriptional Regulation between Tolerant and Sensitive Tomato Genotypes under Cold Stress
Source: PLoS One. 2012 Nov 30;7(11):e50785. doi: 10.1371/journal.pone.0050785 (PMC3511270; doi:10.1371/journal.pone.0050785)
Supplement: Table S4 — Relative expression levels of previously reported cold-responsive genes in the microarray results. (DOC) [file pone.0050785.s008.doc]

**Table S4.** **Relative expression levels of previously reported** **cold-responsive genes in the microarray results.**

| Uingene ID | LA1777 | LA3969 | LA4024 | Annotation | References |
| --- | --- | --- | --- | --- | --- |
| SGN-U213745 | 5.07 | 5.15 | 3.69 | Dehydrin | [45,46] |
| SGN-U214691 | 3.31 | 3.70 | 2.58 | *LeAox1a* | [48] |
| AF385366 | -- | -2.42 | -2.32 | *LeVDE* | [49] |
| SGN-U216769 | 5.76 | 5.83 | 5.07 | *SAP11* | [47] |
| SGN-U219409 | 3.37 | 1.60 | 0.96 | *SAP8* | [47] |
| SGN-U214511 | 3.23 | 3.99 | 3.15 | *TERF2* | [50] |
| SGN-U214427 | -2.22 | -2.15 | -2.02 | *SlGME* | [51] |

Relative expression levels of previously reported cold-responsive genes in the three genotypes at 3 d of cold stress (4 °C). Values are shown as log_2_ stress/control. Data are means of three independent biological replicates. ‘--’ represent the missing values.
