# Supplementary material for: Differential Modulation of Photosynthesis, Signaling, and Transcriptional Regulation between Tolerant and Sensitive Tomato Genotypes under Cold Stress
Source: PLoS One. 2012 Nov 30;7(11):e50785. doi: 10.1371/journal.pone.0050785 (PMC3511270; doi:10.1371/journal.pone.0050785)
Supplement: Table S6 — Significantly enriched GO biological process terms among the differentially expressed genes between tolerant and sensitive tomato genotypes under cold stress. (DOC) [file pone.0050785.s010.doc]

**Table S6. Significantly enriched GO biological process terms among the differentially expressed genes between tolerant and sensitive genotypes under cold stress.**

| **Gene Ontology term** | **Cluster frequency** | **Genome frequency of use** | **P-value** |
| --- | --- | --- | --- |
| response to stimulus | 38 out of 92 genes, 41.3% | 2636 out of 12672 genes, 20.8% | 0 |
| response to stress | 28 out of 92 genes, 30.4% | 1771 out of 12672 genes, 14.0% | 0.02 |
| response to chemical stimulus | 24 out of 92 genes, 26.1% | 1499 out of 12672 genes, 11.8% | 0.03333 |
| response to abiotic stimulus | 16 out of 92 genes, 17.4% | 962 out of 12672 genes, 7.6% | 0.048 |
| response to endogenous stimulus | 15 out of 92 genes, 16.3% | 830 out of 12672 genes, 6.5% | 0.04273 |
| response to light stimulus | 10 out of 92 genes, 10.9% | 390 out of 12672 genes, 3.1% | 0.04667 |
| response to radiation | 10 out of 92 genes, 10.9% | 401 out of 12672 genes, 3.2% | 0.04421 |
| response to auxin stimulus | 8 out of 92 genes, 8.7% | 312 out of 12672 genes, 2.5% | 0.04483 |
| fatty acid biosynthetic process | 6 out of 92 genes, 6.5% | 146 out of 12672 genes, 1.2% | 0.042 |
| response to UV | 6 out of 92 genes, 6.5% | 117 out of 12672 genes, 0.9% | 0.025 |
| response to reactive oxygen species | 6 out of 92 genes, 6.5% | 122 out of 12672 genes, 1.0% | 0.02714 |
| response to heat | 6 out of 92 genes, 6.5% | 123 out of 12672 genes, 1.0% | 0.02533 |
| response to gibberellin stimulus | 5 out of 92 genes, 5.4% | 116 out of 12672 genes, 0.9% | 0.04308 |
| oxylipin metabolic process | 5 out of 92 genes, 5.4% | 42 out of 12672 genes, 0.3% | 0 |
| phytosteroid metabolic process | 4 out of 92 genes, 4.3% | 24 out of 12672 genes, 0.2% | 0.035 |
| brassinosteroid metabolic process | 4 out of 92 genes, 4.3% | 24 out of 12672 genes, 0.2% | 0.028 |
| steroid metabolic process | 4 out of 92 genes, 4.3% | 65 out of 12672 genes, 0.5% | 0.04783 |
| flavonol metabolic process | 3 out of 92 genes, 3.3% | 15 out of 12672 genes, 0.1% | 0.03 |
| heat acclimation | 3 out of 92 genes, 3.3% | 13 out of 12672 genes, 0.1% | 0.0325 |
| long-day photoperiodism | 2 out of 92 genes, 2.2% | 4 out of 12672 genes, 0.0% | 0.0325 |
| calcium-mediated signaling | 2 out of 92 genes, 2.2% | 6 out of 12672 genes, 0.0% | 0.04286 |

The analysis was performed with the 92 differentially expressed genes between tolerant and sensitive genotypes using the Tomato Functional Genomics Database with a P value cutoff of less than 0.05 [32].
